# Numerical modelling of downstream scour in circular culverts: Impact of inlet blockages and variable flow conditions

**DOI:** 10.1371/journal.pone.0312501

**Published:** 2024-10-31

**Authors:** Kaywan Othman Ahmed, Mohammad Reza Kavianpour, Ata Amini, Younes Aminpour

**Affiliations:** 1 Department of Civil Engineering, K. N. Toosi University of Technology, Tehran, Iran; 2 Civil Engineering Department, Tishk International University, Sulaimani, Iraq; 3 Department of Civil Engineering, K.N. Toosi Univ. of Technology, Tehran, Iran; 4 Kurdistan Agricultural and Natural Resources Research and Education Center, AREEO, Sanandaj, Iran; 5 Department of Hydraulic and Hydro-Environmental Engineering, Water Research Institute, Ministry of Energy, Tehran, Iran; Ardakan University, ISLAMIC REPUBLIC OF IRAN

## Abstract

An accurate estimate of scour depth downstream of culvert outlets is essential for culvert design integrity. Inadequate designs can result in structural failures, leading to increased costs for maintenance and rehabilitation. The present research evaluates the efficacy of numerical models in predicting scour depth and its location downstream of circular culverts under variable flow conditions. Two hydrographs were created for unsteady flow, featuring nine different flow discharges, while steady flow conditions were analysed at flow rates of 14 *l/s* and 22 *l/s*. The study investigated circular culverts with inlet blockages of 0%, 15%, and 30%, comparing outcomes with predictions from the Flow-3D software using the renormalisation group (RNG) turbulence model. Extensive experimental data on circular culverts were utilised, with simulations performed using commercial software. This involved analysing the scour’s downstream profile, its maximum depth, and its location, and comparing these metrics with actual observed data. The results revealed that the numerical model predictions closely corresponded to the experimental data, even though the simulated scour was generally less than that observed for steady and unsteady flows. The results showed that in unsteady flow conditions and for the discharge of 22 *l/s*, 30% blockage increased scour by 6.8% and 14.2%, respectively, compared to 15% blockage and non-blocked flow. This increase was 22% and 9.5% for the discharge of 14 *l/s*, respectively. In the steady case, when the flow rate was adjusted from 14 *l/s* to 22 *l/s*, there was a noticeable increase in scour depth downstream of the culvert. While blockage rates impacted the scour patterns significantly in unsteady flow scenarios, escalating blockage percentages did not lead to uniformly proportional increases in scour depth within steady flow environments.

## 1 Introduction

A culvert acts as an essential component in hydraulic engineering, playing a key role in controlling water movement and determining flow rates for water traveling over surfaces, such as those found in streams and rivers. It is indispensable for facilitating the flow of local drainage through and under man-made structures like road embankments and various types of water crossing infrastructures. Typically short, these engineered channels are designed to efficiently divert water across physical obstacles such as railways, highways, and roads. In recent decades, to improve the accuracy of predicting scour depths, a vital consideration in both the design and upkeep of these structures has focused on creating empirical formulas in much of the research carried out. These formulas are primarily derived from systematic observations in laboratory settings and seek to offer precise predictions of scour depths across different hydraulic settings [1, 2].

These studies have primarily concentrated on grasping the greatest depth of scour and the enlargement of scour hole sizes. Choosing the right geometry for scour holes and types of protective measures is crucial for designing culverts in a manner that is both safe and economically viable across various flow hydrographs. Furthermore, previous research has delved into the patterns of scour and the deepest points reached, both across and along the direction of flow at culvert sites.

Research conducted by [3] explored the effects of turbulent flow patterns on scouring at the exits of culverts, focusing on aspects such as turbulence intensity, average speeds, structures of bursting near the bed, and Reynolds stresses. Importantly, [4] collected data during significant storm events on how debris can obstruct culverts and bridge openings, highlighting the importance of the apparent size of the opening in determining the extent of the blockage.

In their investigation into the changes in both morphology and water dynamics around culvert structures, [5] conducted experiments to analyze the characteristics of turbulence and the velocity of water approaching culverts from upstream. This study shed light on the intricate behaviors of water flow, particularly in the context of designing culverts with multiple barrels, underscoring the necessity to account for complex flow dynamics in practical engineering applications. Expanding on this theme, [6] engaged in detailed scouring experiments focused on the conditions at the culvert outlet, the properties of the surrounding sediments, and the physical form of the culverts themselves. Their findings illustrated a direct linear relationship between the maximum depth of scour relative to the hydraulic radius and the particle densimetric Froude number, particularly in scenarios where the culvert outlets were either completely unobstructed or partially blocked. Further enriching this area of study, [7] embarked on an exhaustive experimental examination to pinpoint the factors with the most significant impact on scouring near culverts. They highlighted the critical roles played by structural features such as wing walls and floor slabs at the inlets and outlets as well as operational conditions such as the level of blockage and the depth of water downstream in conjunction with the culvert’s design shape in influencing the occurrence and severity of local scour around these hydraulic structures.

Under unsteady flow conditions, [1] conducted detailed experiments on the scour processes at box culverts, focusing on both obstructed and unobstructed conditions. Their research unveiled that a significant portion, ranging from 88% to 98% of the deepest scour, occurred in the ascending phase of the hydrograph. On a related note, [8] investigated the impact of different levels of inlet blockage and submersion on the performance of culverts and the depth of scour holes under varied flow intensities. Their observations revealed that at a constant flow rate, an increase in the submersion level led to a reduction in the maximum depth of scour. In contrast, scenarios with inlet obstruction showed that a higher ratio of blockage was linked to an increase in the maximum depth of scour relative to the flow conditions. The results proved that the blockage ratio of 70% of culvert height makes the water depth upstream increase by 2.3 times of culvert height, and mean velocity increases by 3 times more than in the base case.

Although predicting scour depth at culvert outlets with empirical models has been challenging due to the complexity of flow and sediment dynamics, the evolution of computational fluid dynamics (CFD) such as Flow-3D software has introduced new opportunities for accurately assessing scour depth and its driving mechanisms. The application of numerical approaches via Flow-3D has proven effective, as demonstrated by research that aligns numerical simulations closely with results from laboratory experiments. [9] investigated the effects of the ratio of partial blockage on scour processes downstream of box culverts under unsteady flow conditions, discovering a significant alignment between the results from laboratory experiments and numerical simulations via Flow-3D. Numerical simulations show more maximum scour depth than experimental data, with about 20% more for non-blocked cases and 32% more for 40% inlet blockage. Similarly, [10] assessed the efficacy of numerical models in forecasting scour depths downstream of box culverts under variable flow conditions, finding a reliable match between the predictions made by numerical models and actual experimental outcomes. Numerical simulations indicate higher maximum scour depths compared to experimental data. For non-blocked cases, the data differed by 16.8%, and for 40% blockage, the difference was 29%. Their analysis illustrated that in both turbulence models studied (RNG and k-ε), an increase in water discharge during the hydrograph’s upward trend led to an increase in scour depth, which then decreased with reduced discharges during the downward trend.

[11] explored how well numerical models could predict the scour depth found downstream from a box culvert, utilizing Flow-3D software in conjunction with surrogate models developed through the Box-Behnken technique. They examined various design-related factors of culverts and observed a good agreement between the predictions of the surrogate models and the numerical analyses. The shortage of experimental data concerning unsteady flow conditions necessitates in-depth simulations that accurately depict the flow and scouring behaviors downstream of culverts in fluctuating flow scenarios.

This study introduces several key innovations that distinguish it from previous investigations. Unlike the traditional focus on box culverts, this research employs circular culverts and examines blockage rates of 0%, 15%, and 30%, which differ from the commonly used 0%, 20%, and 40% rates. The study also utilizes the new Flow-3D version 11.0.4, and it develops two distinct hydrographs for unsteady and steady flow conditions, incorporating novel flow rates and step durations, in contrast to the single unsteady hydrograph typically used in prior studies. The research further explores the prediction of scour depth and its specific location for circular culverts, both unblocked and partially blocked, under varying flow conditions. By leveraging experimental data from open culverts, the study evaluates the performance of different turbulence models within the software, offering a comprehensive comparison between computational predictions and experimental observations. This approach provides new insights into the modeling of complex hydraulic processes involving circular culverts.

## 2 Material and methods

### 2.1 Numerical model

In this study, which employs Computational Fluid Dynamics (CFD), the numerical model software Flow-3D was utilized, and the result was compared to the observed data by [12]. CFD has emerged as a prominent method for flow analysis, particularly when compared to experimental approaches [9]. The impact of surface tension is incorporated into the Navier–Stokes equation through a pressure gradient term. Specifically, the pressure term is partitioned into two components: one dedicated to achieving mass conservation and the other addressing surface tension effects. This partitioning allows for the preservation of surface tension-induced pressure conditions at the free surface [13].

This study utilized the Reynolds-Averaged Navier-Stokes (RANS) equations for hydrodynamic modeling and employed the Volume of Fluid (VOF) technique to track water surfaces and solve nonlinear Navier-Stokes equations, particularly in 3D flow and liquid motion scenarios [14]. The Fractional Area/Volume Obstacle Representation (FAVOR) method was applied to delineate domain boundaries and flow obstacle zones. The VOF function’s non-uniform distribution at the free surface requires a reconstruction procedure for assessing flux across cells [15]. The Fractional Volume method was used to locate fluid surfaces within grid elements [16].

### 2.2 Governing equations

#### 2.2.1 Reynolds Averaged Navier-Stokes

For three-dimensional analysis of an incompressible fluid state within the Flow-3D software, the Reynolds-Averaged Navier-Stokes (RANS) equations in the Cartesian coordinate system were employed as outlined by [17]:

$$\frac{\partial }{\partial x_{i}}(u_{i}A_{i})=0$$ (1)

$$\frac{\partial u_{i}}{\partial t}+{\frac{1}{V}}_{F}(u_{j}A_{j}\frac{\partial u_{i}}{\partial x_{j}})=-\frac{1}{\rho }\frac{\partial p}{\partial x_{i}}+G_{i}+f_{i}$$ (2)

where *t* = time, *u*_*i*_ = mean velocity, *V*_*F* =_ fractional volume open to flow, *x* = the coordinate, *A*_*i*_ = fractional area open to flow, *G*_*i*_ = the body accelerations, *ρ* = fluid density, *f*_*i*_ = the viscous accelerations, *p* = pressure, and the subscripts *i* and *j* refer to the Cartesian coordinate directions (x, y, z).

#### 2.2.2 Turbulence model

Within Flow-3D modelling, turbulent flow is addressed through five schemes: Prandtl’s mixing length theory, Large Eddy Simulation (LES), Renormalization-Group (RNG), one-equation turbulent energy (*k*), and the two-equation (*k-ε*) models as detailed in [16]. Notably, the RNG model, with equations resembling those in the *k-ε* model, is distinctive for its precision in characterizing flows with robust shear regions and low-intensity turbulence. While air entrainment is assumed to stem from turbulent disturbances at a free surface, for effective turbulence modelling, preference is given to the k-ε or RNG models. In scenarios involving non-laminar flow, as commonly encountered in scour-type simulations, the RNG model is recommended as the primary choice [16].

In this study, both RNG turbulence models were employed for Computational Fluid Dynamics (CFD) simulations using Flow-3D. Noteworthy developments include the recent reformulation of the RNG model. The turbulence eddy, *v*_*t*_, based on the Boussinesq approximation, plays a pivotal role in solving the turbulence equation [18].

$$v_{t}=c_{u}\frac{k^{2}}{\varepsilon }$$ (3)

where *k* and *ε* are turbulence kinetic energy and dissipation, respectively and *C*_*μ*_ = an empirical coefficient. In the standard *k-ε* turbulence model, both *k* and *ε* are defined by Eqs (4) and (5) [17]:

$$\frac{\partial k}{\partial t}+u_{i}\frac{\partial k}{\partial x_{i}}=\frac{\partial }{\partial x_{i}}\left(\frac{v_{t}}{\sigma_{k}}\frac{\partial k}{\partial x_{i}}\right)+v_{t}\left(\frac{\partial u_{i}}{\partial x_{j}}+\frac{\partial u_{j}}{\partial x_{i}}\right)\frac{\partial u_{i}}{\partial x_{j}}-\varepsilon$$ (4)

$$\frac{\partial \varepsilon }{\partial t}+u_{i}\frac{\partial k}{\partial x_{i}}=\frac{\partial }{\partial x_{i}}\left(\frac{v_{t}}{\sigma_{\varepsilon }}\frac{\partial \varepsilon }{\partial x_{i}}\right)+C_{1}\frac{\varepsilon }{k}v_{t}\left(\frac{\partial u_{i}}{\partial x_{j}}+\frac{\partial u_{j}}{\partial x_{i}}\right)\frac{\partial u_{i}}{\partial x_{j}}-C_{2}\frac{\varepsilon^{2}}{k}$$ (5)

where *C*_*1*_ and *C*_*2*_ with corresponding values of 1.44 and 1.92 act as constants in adjusting turbulence dissipation and generation within the *ε* equation. The RNG model suggests empirical constants *σ*_*k*_ = 1.0 and *σ*_*ε*_ = 1.3 [19]. Taking into consideration the non-flat topography of riverbeds in real-world applications, particularly in the context of sloping surfaces, the critical Shield parameter is modified related to the angle of repose and is presented in Eq (6) according to [20].

$$\theta^{'}=\theta_{cr}\frac{\cos\psi \sin\beta \sqrt{\cos^{2}\beta \tan^{2}\varphi -\sin^{2}\psi \sin^{2}\beta }}{\tan\varphi }$$ (6)

where *θ’* signifies the critical Shields’ parameter for the onset of particle motion, *β* represents the slope bed angle, *φ* indicates the sediment’s angle of repose, and *ψ* denotes the angle between the upslope direction and the flow. The calculation of the local bed shear stress (*τ*) is determined by Eq (7):

$$\tau =\theta gd(\rho_{s}-\rho )$$ (7)

Where *ρ*_*s*_ = the sand density, *ρ* = the density of fluid, *d* = the particle diameter, and *θ* = the Shields’ parameter. The dimensionless bed load transport rate for sediment, as formulated by Meyer-Peter and Muller, is applicable using Eq (8) as detailed by [21].


$$\Phi ={\left(\theta -{\theta^{'}}_{cr}\right)}^{1.5}$$ (8)

The calculation of the volumetric bed-load transport rate (*q*_*b*_) is determined using Eq (9).

$$q_{b}=\Phi {\left[g(\frac{\rho_{s}-\rho }{\rho })d^{3}\right]}^{\frac{1}{2}}$$ (9)

where *d* is the sediment diameter, and in the case of turbulent association, the Shallow Water Flow is applied. Eq (10) is employed to estimate the drag force coefficient concerning flow depth and surface roughness [17]:

$$C_{d}={\left[\frac{\kappa }{B+\ln(\frac{k_{s}}{30y_{0}})}\right]}^{2}$$ (10)

where *k* denotes the Von Karman constant (*k* = 0.40), *B* is a constant set at 0.71 according to [17], *y*_*0*_ represents the fluid depth, and [22] assumed *k*_*s*_ as the surface roughness with *k*_*s*_ = 2*d*_*50*_ as specified in [16].

The Volume of Fluid (VOF) and Fractional Area Obstacle Representation Method (FAVOROTM) exemplify volume fraction methodologies. In these techniques, the region is initially subdivided into control volumes or smaller grid elements. An effective means of characterizing element states involves introducing a parameter *F*, which quantifies the fraction of the element’s volume filled with liquid as detailed in [16]. This function represents the VOF per unit volume and adheres to the following equations:

$$\frac{\partial F}{\partial t}+\frac{1}{V_{F}}\left[[\frac{\partial }{\partial x}(FA_{x}u)+R\frac{\partial }{\partial y}(FA_{y}v)+\frac{\partial }{\partial_{z}}(FA_{z}w)+\xi \frac{FA_{x}u}{x}\right]=F_{DIF}+F_{SOR}$$ (11)

$$F_{DIF}=\frac{1}{V_{F}}\left\{\frac{\partial }{\partial x}(v_{F}A_{x}\frac{\partial F}{\partial x})+R\frac{\partial }{\partial x}(v_{F}A_{y}R\frac{\partial F}{\partial x})+\frac{\partial }{\partial z}(v_{F}A_{z}\frac{\partial F}{\partial z})+\xi \frac{v_{F}A_{x}F}{x}\right\}$$ (12)

$$V_{F}\frac{\partial \rho }{\partial t}+\frac{\partial }{\partial x}(\rho uA_{x})+R\frac{\partial }{\partial y}(\rho vA_{y})+\frac{\partial }{\partial z}(\rho wA_{z})+\xi \frac{\rho uA_{x}}{x}=R_{DIF}+R_{SOR}$$ (13)

In this theoretical framework, *F*_*DIF*_ delineates a diffusion term specifically designed for the intricate process of two-fluid mixing. *F*_*SOR*_ signifies the temporal variation of the volume fraction of fluid, intimately linked to the mass source and representing the fractional volume accessible to the flowing medium. *R*_*SOR*_ is identified as a source of mass, while *R*_*DIF*_ is designated as a term accounting for turbulent diffusion. The fractional area open to flow is symbolized as *A*_*x*_ in the x-direction, while *A*_*y*_ and *A*_*z*_ portray analogous area fractions for fluid flow in the *y* and *z* directions. The velocity components (*u*, *v*, w*)* are aligned with the coordinate directions (*x*, *y*, z*)* or represented as (*r*, *R*_*SOR*_, *z*), ensuring consistency in the context of spatial orientation.

### 2.3 The data

#### 2.3.1 Experimental setup

This study used experimental data to assess scour at the outlet of a culvert conducted at the Hydraulics Laboratory of the University of Sulaimani (UoS) in Kurdistan, Iraq. Among the limitations of the present research are the laboratory conditions. In this research, the geometrical parameters of the culvert, including width, length, and longitudinal slope, was considered constant. Also, the width of the channel was fixed. A Perspex flume, measuring 7.9 m in length, 0.7 m in depth, and 0.6 m in width, was utilized with a consistent bed slope of 0.001 and a water recirculation mechanism. The culvert inlet was positioned 4.6 m from the flume inlet to ensure a fully developed flow in the test area. The experimental setup included a sand basin, 4.4 m in length, 0.15 m in depth, and matching the flume’s width. The sands exhibited a median grain size of *d*_*50*_ = 1.1 mm and a geometric standard deviation of *σ*_*g*_ = 2.9 mm, categorizing them as uniform sediment [23, 24]. The sediment was appropriately graded to match the culvert inlet and outlet elevations, and glass material was used for inlet blockages.

This research adhered to *Re* > 10^5^ to reduce scale effects on scour depth in agreement with [25] and acknowledged that viscous effects diminish beyond *Re* > 10^4^. The study accounted for the flume width’s influence on scour by maintaining a culvert-to-flume width ratio of 1:3, reflecting insights from [26]. It consistently used fixed culvert dimensions to lessen contraction impacts on scour, focusing on partially full flows and normalized scour depths to gauge effects accurately.

#### 2.3.2 The numerical cases

**Fig 1:**
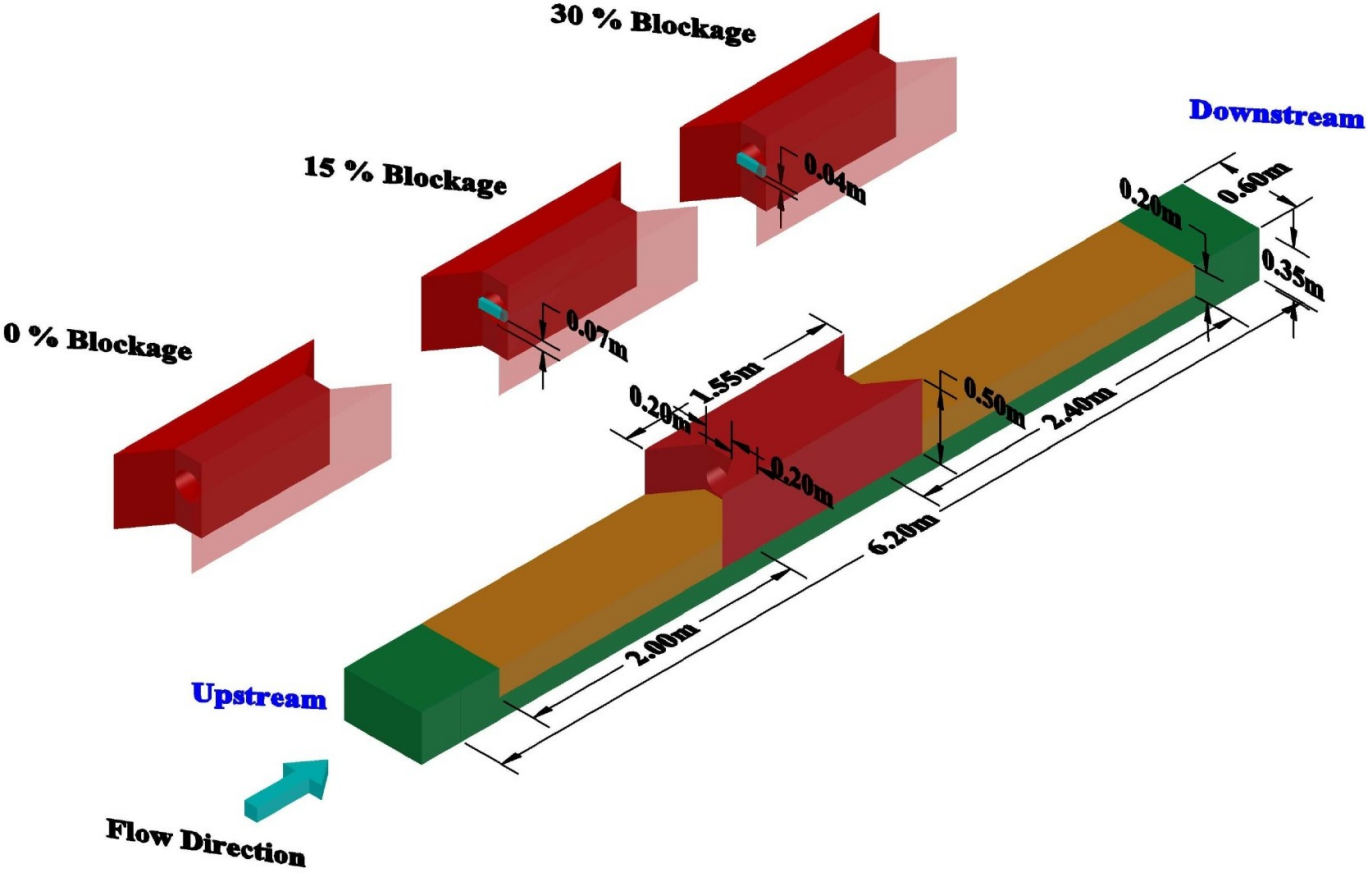
Detailed view of culvert with blockage rates.

In this study, the Flow-3D software version 11.0.4 was used to accurately model experimental scenarios that are currently under peer review. The present study, aimed to bridge the gap between computational predictions and pending experimental outcomes, providing preliminary insights and validations of the simulated models against expected experimental data. This study employed a circular culvert shape, with a barrel length of 1 m and an opening diameter of 0.2 m as shown in Fig 1. To enhance flow stability, both the culvert’s outlet and inlet featured 30° flare transitions [27]. A symmetric hydrograph was adopted to facilitate the conveyance of unsteady flow through the culvert. Under unsteady flow conditions, two different hydrographs were created, and the maximum flow rates from each hydrograph were used as steady cases for the equivalent duration. The hydrograph was generated for both unsteady and steady clear water flow scenarios, comprising nine distinct flow discharges. The duration of each step in the first hydrograph (*Q*_*m*_ = 22 *l/s*) was 40 minutes, while the second hydrograph (*Q*_*m*_ = 14 *l/s*) spanned 25 minutes. In cases of partial blockage, the submerged upstream and downstream areas of the culvert could induce a transition from supercritical to subcritical flow near its terminus. The culvert’s capacity determined the maximum flow in the hydrograph. Table 1 provides comprehensive details on the numerical cases conducted in this research.

**Table 1:** Numerical cases.

Model	Flow Conditions	Maximum Discharge *Q*_*m*_ (*l/s*)	Blockage (%)	Time (min)
Circle	Unsteady	22	0	360
			15	360
			30	360
		14	0	225
			15	225
			30	225
	Steady	22	0	360
			15	360
			30	360
		14	0	225
			15	225
			30	225

The values of critical shear velocity ($u_{{*}_{c}}$) and critical flow velocity (*V*_*c*_) were presented based on the equations proposed by [23]. These equations are as follows:

$$u_{{*}_{c}}=0.0305d_{50}^{0.5}-0.0065d_{50}^{-1}$$ (14)

$$\frac{V_{c}}{u_{{*}_{c}}}=5.75\;\log\left[5.53\frac{y_{w}}{d_{50}}\right]$$ (15)

where *y*_*w*_ is the water depth. The critical shear velocity, for all cases, depends on the sediment size (*d*_*50*_) and flow depth. In all the test the critical shear was more than the shear velocity and clear water condition was achieved

#### 2.3.3 Model scale

In the context of the model scale, experiments with small-scale models, as noted by [28], often fail to precisely calculate maximum scour depth, yielding unreliable results. Conversely, large-scale experiments can incur high laboratory costs and facility requirements and might also lead to contraction scour. To mitigate scale effects on scour depth, this research specifically utilized data with Reynolds number *R*_*e*_ >10^5^, following the approach outlined by [25]. It is noteworthy that beyond *R*_*e*_ >10^4^, viscous effects become insignificant, making the Reynolds number a critical factor for reliable results, as emphasized by [28].

#### 2.3.4 Culvert geometry and grid construction

**Fig 2:**
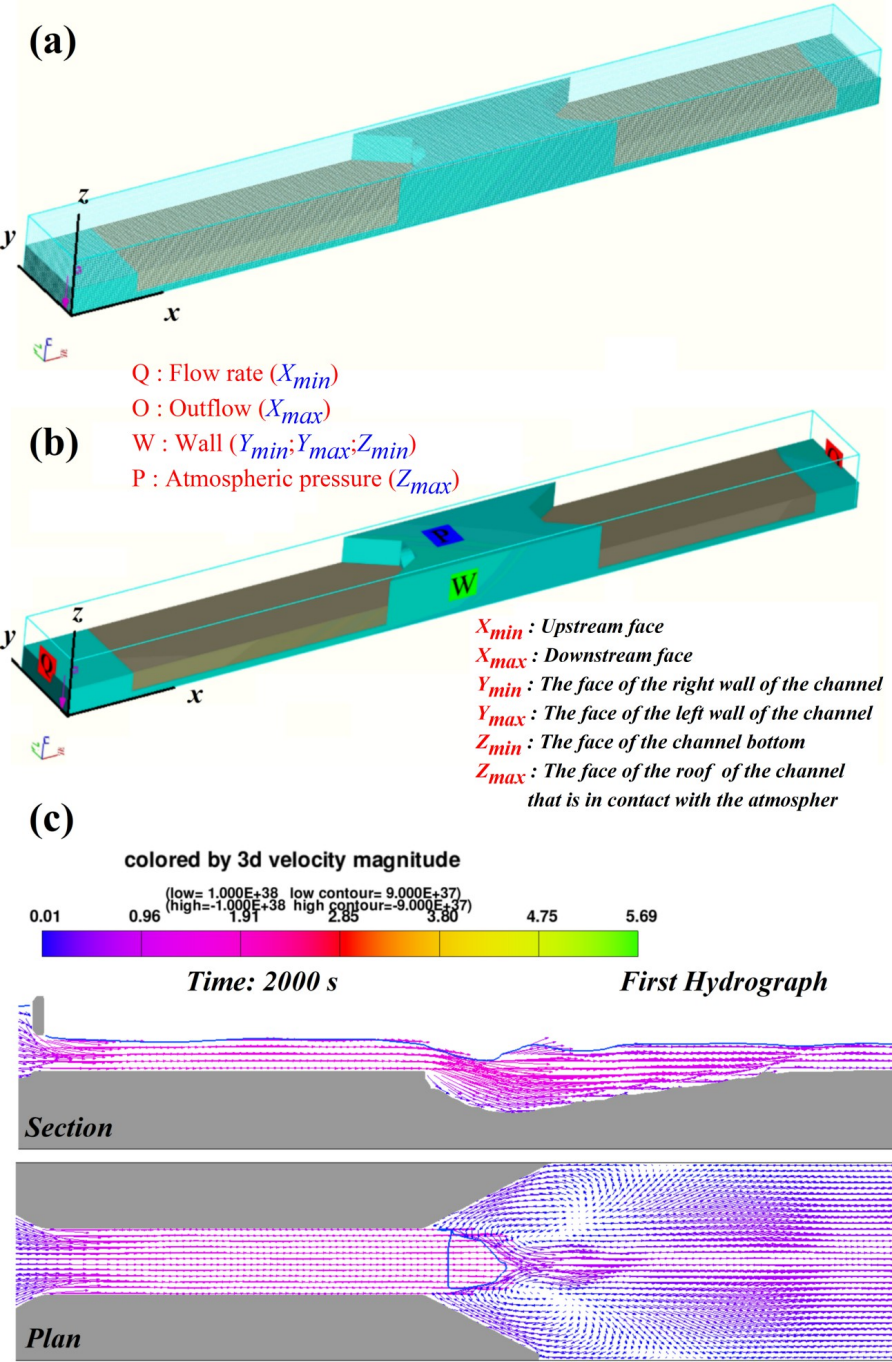
Depiction of a) the model coordination and geometry meshing. b) boundary conditions. c) Flow visualization with the vortex’s direction and streambed degradation.

In utilization of unstructured meshes in a classical culvert setting, typically characterized by a horizontal rectangular channel, becomes particularly advantageous in regions where large gradients of flow parameters are expected. This allows for selective refinement of specific areas, as noted by [29]. While acknowledging the generally lower precision of unstructured meshes, emphasized by [30, 31], their algorithms contribute to reduced latency in simulations and ensure more regular memory access [32]. In the context of multiphase flows, the use of topologically orthogonal meshes tends to minimize numerical issues. Hence, in this study, a structured rectangular hexahedral mesh was deemed more suitable for the problem. Mesh generation, facilitated by the FAVOR technique with orthogonal meshes defined in Cartesian coordinates in Flow-3D, was employed for the complex model shapes. Through trial and error, the mesh dimension of 0.015 m mesh size was decided upon in the x, y, and z directions to strike a balance between the total number of cells and the desired quality and accuracy in the results. Fig 2 illustrates the model coordination and geometry meshing.

#### 2.3.5 Boundary conditions

The governing equations for fluid flow motion fall under the category of initial boundary values. This implies that the solution must be determined at both the initial time and the boundary. In establishing the scour model, Fig 2 illustrates the boundary conditions, where a specified flow rate is set at the inlet, the outflow is set at the outlet, and the top side is designated with a specified pressure. The right and left sides, in addition to the bottom side are defined as wall boundaries [9].

#### 2.3.6 Flow pattern and scour formation

The prediction of localized scour geometry at the culvert outlet is a crucial element in the culvert design process, guiding decisions on erosion protection. Fig 2 presents both a 3D and side view of the streambed, providing insights into scour formation and flow interaction at the culvert outlet under a flow rate of 10 *l/s*, utilizing the RNG equation. The observed direction of vortices contributes to an initial understanding of the flow structure within scour holes. Notably, as the scour phenomenon intensifies, an increase in vortices at these locations occurs, influencing the overall flow rotation appearance.

#### 2.3.7 Error measurement

In this research, the accuracy of the numerical model was rigorously assessed using various statistical indicators. To accomplish this, the simulated values of scour depth were compared with the corresponding experimental data. These comparative analyses enabled to identify how closely our model’s predictions aligned with observed results from experimental tests. The correlation coefficient (*R*^*2*^), root mean square error (RMSE), mean absolute error (MAE) and mean absolute percentage error (MAPE) represented by Eqs (14)–(17) were used to model the evaluation [33].

$$R^{2}=\frac{{\left[\sum_{i=1}^{n}\left(P_{i}-\bar{P})(O_{i}-\bar{O}\right)\right]}^{2}}{\sum_{i=1}^{n}{\left(P_{i}-\bar{P}\right)}^{2}\sum_{i=1}^{n}{\left(O_{i}-\bar{O}\right)}^{2}}$$ (16)

$$RMSE=\sqrt{\frac{1}{n}\sum_{i=1}^{i=n}{\left(P_{i}-O_{i}\right)}^{2}}$$ (17)

$$MAE=\frac{1}{n}\sum_{i=1}^{n}\left|p_{i}-O_{i}\right|$$ (18)

$$MAPE=\frac{1}{n}\sum_{i=1}^{n}\left|\frac{p_{i}-O_{i}}{P_{i}}\right|$$ (19)

where *n*, *P*_*i*_, *O*_*i*_, $\bar{P}$ and $\bar{O}$ are respectively the number of data, the value of scour depth or location in the numerical model, the corresponding observed values, the average numerical model data, and the average observational data. The value of *R* is always between -1≤ *R* ≤1 and as *R* was close to +1, the accuracy of the model was optimal. To measure the confidence of the model’s prediction of the observed data, the MAE was used as a common indicator. The sample standard deviation of the differences between simulated values and experimental data is presented by RMSE. The lower the MAE and RMSE values, the better the model performance. The MAPE calculates the average absolute error to show the accuracy of a forecast system.

## 3. Result and discussion

### 3.1 Unsteady flow conditions

Using two distinct hydrographs for circular culverts, the effects of discharge on scour were illustrated. This section outlines the test results conducted with a hydrograph featuring *Q*_*M*_ = 22 *l/s* as the first hydrograph and *Q*_*M*_ = 14 *l/s* as the second hydrograph.

#### 3.1.1 The first hydrograph

**Fig 3:**
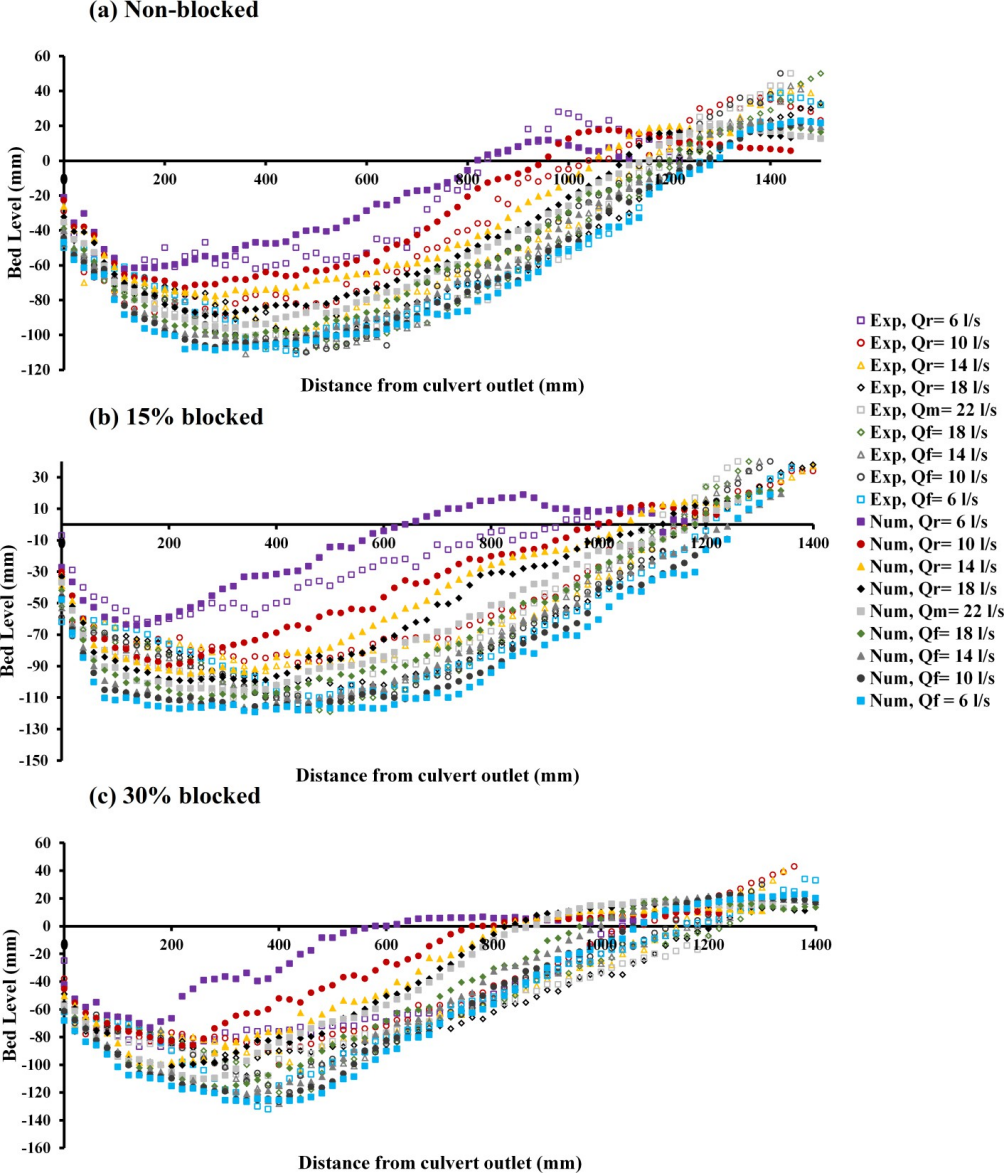
Scour profile for the first hydrograph: a) non-blocked, b) 15% blocked, and c) 30% blocked. (*r*, *m*, and *f* represent raising limb, maximum flow rate, and falling limb of the hydrograph, respectively).

A study was conducted to confirm the precision of the numerical model through a comparison between experimental observations of bed scour and the outcomes from numerical simulations. Fig 3 illustrates the formation of scour profiles for both experimental and numerical data downstream of the circular culvert, blocked or unblocked, at various flow rates under conditions of unsteady flow achieved through the application of RNG.

Fig 3(A) displays the case of a completely open culvert, where the flow rate begins at *Q*_*r*_ = 6 *l/s*, climbs to a peak of *Q*_*m*_ = 22 *l/s*, and subsequently decreases back to *Q*_*f*_ = 6 *l/s*. In this illustration, both the observed and numerical data at the initial flow rate of *Q*_*r*_ = 6 *l/s* demonstrates sufficient flow to displace sediment. The deepest scour observed experimentally was near 0.066 m, compared to the numerical finding of approximately 0.0623 m, suggesting a reduction in the observed data’s culvert change rate by approximately 5.6%. The increase in scour depth, as noted in both the experimental and numerical data, corresponded with each incremental rise in discharge rate. The observed data identified the largest scour depth at a reduced flow rate of *Q*_*f*_ = 14 *l/s*, reaching approximately 0.111 m, whereas the numerical data observed its maximum at a decreased flow rate of *Q*_*f*_ = 6 *l/s*, marking a depth close to 0.108 m. This outcome indicates that the maximum scour depth measured experimentally was 2.7% greater than that determined numerically.

Fig 3(B) depicts the change in scouring due to a blockage at the inlet of 15%. A comprehensive examination of the scour profiles indicated that at the outset of the stepwise analysis, there was a notable similarity between the data obtained from experiments and that derived from numerical simulations. As the analysis proceeds through subsequent stages, a divergence was observed; the experimental data began to show a considerable deviation from the initial results, while the numerical data exhibited consistency and alignment with the ongoing changes in scour patterns. This observation highlights the complexities involved in accurately capturing the dynamics of scouring processes and underscores the importance of integrating both experimental and computational approaches for a thorough understanding. Within the observed data, the continuity of maximum scour depth progressively rises until reaching a *Q*_*f*_ = 18 *l/s*, after which it slightly declines. Conversely, in numerical data, this measure continues to ascend steadily up until the final phase of the hydrograph at *Q*_*f*_ = 6 *l/s*. For both the observed and the simulated data, the peak scour depth was noted at a flow rate decline, with the values reaching approximately 0.119 mm at *Q*_*f*_ = 18 *l/s* and approximately 0.118 mm at *Q*_*f*_ = 6 *l/s*, respectively. The data showed a variation rate of approximately 0.84% when comparing the observed data and the numerical data.

Fig 3(C) illustrates the scour pattern resulting from a 30% blockage at the inlet. In this scenario, both the experimental and numerical data indicate an increased level of scour at the culvert’s outlet when compared to prior instances. The size of the scour hole showed a direct correlation with variations in flow rate. Similarly, to the 15% blockage scenario, the depth of scour for the 30% blockage also exhibited greater similarity in later steps than in the initial step for experimental data, whereas the numerical data-maintained consistency and closely followed the changes in scour patterns as the flow rate increased. The deepest scour recorded, both in the observed and numerical data, was at the end of the hydrograph with a *Q*_*f*_ = 6 *l/s*, measuring 0.132 m and 0.127 m, respectively.

The overall dataset revealed that scour occurrences were more pronounced in the experimental data than in the numerical data. Concerning maximum scour depth, the simulation data showed that a 30% obstruction led to an increase of approximately 6.8% and 14.2%, relative to conditions of a 15% blockage and an unobstructed flow, respectively. This indicates a notable enhancement in the progression of the scouring activity [9, 10].

#### 3.1.2 The second hydrograph

**Fig 4:**
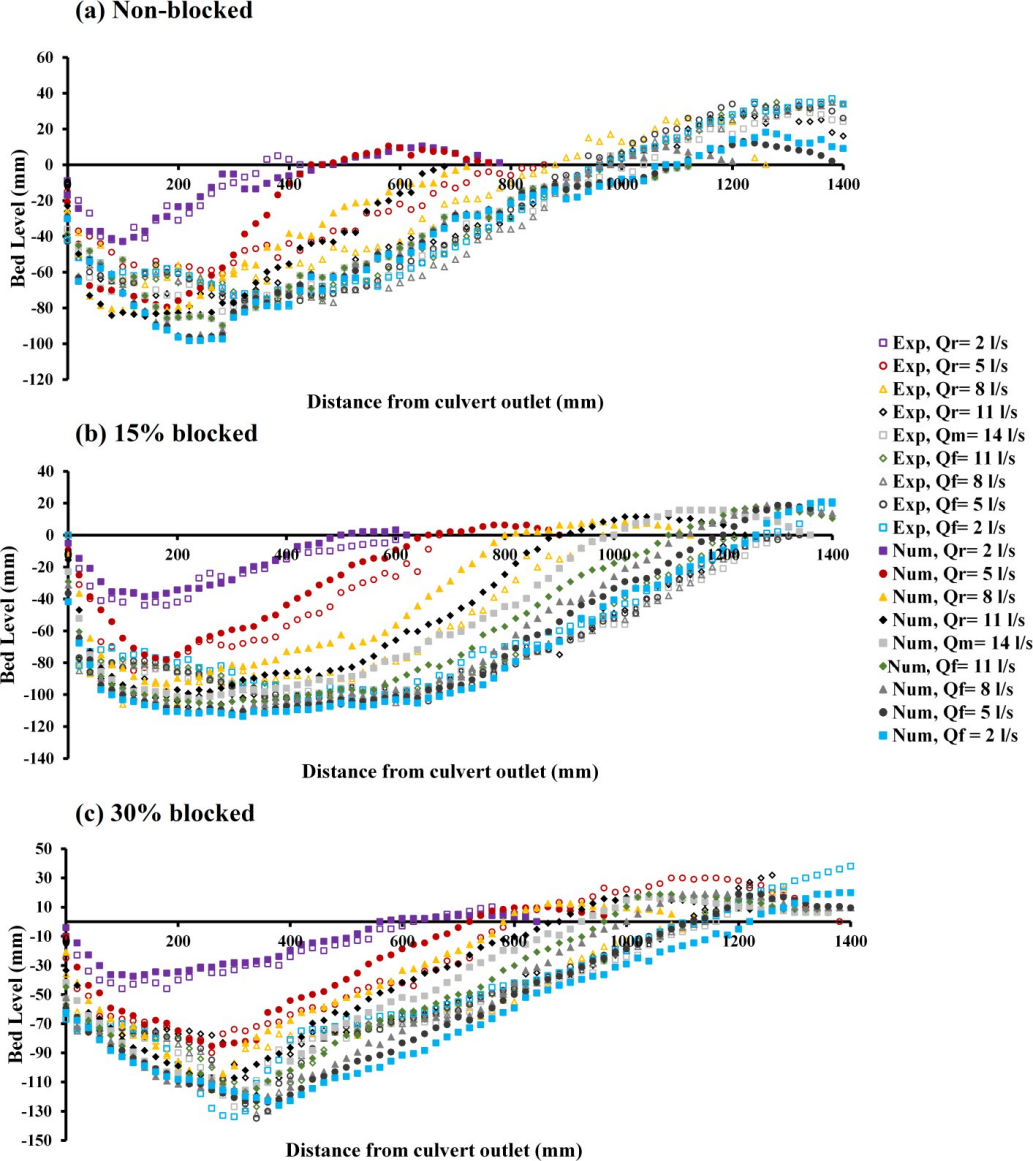
Scour profile for the second hydrograph: a) non-blocked, b) 15% blocked, and c) with 30% blocked.

Fig 4 presents the variation of the scour profile for observed and numerical data at the circular culvert outlet under unsteady flow condition*s*. The analysis considered experiments with both nonblocked and blocked cases. The second hydrograph has a symmetrical pattern, commencing at a flow rate of *Q*_*r*_ = 2 *l/s*, reaching a peak rate of *Q*_*m*_ = 14 *l/s*, and declining to *Q*_*f*_ = 2 *l/s*.

Fig 4(A) illustrates the scour pattern observed in the case of a culvert that is fully open. Upon examining the data across various stages of the hydrograph, a comparison between the observed and numerical datasets reveals that the profiles of scouring are remarkably similar to one another. This similarity suggests a consistent correlation between the experimental observations and the numerical simulations throughout the different phases of the hydrograph analysis.

At the initial stage of the hydrograph analysis, an increase in scour profiles is evident in both observed and simulated data, coinciding with a rise in flow rate. The experiment recorded the maximum scour depth at a flow rate of *Q*_*m*_ = 14 *l/s*, achieving roughly 0.08 m. Conversely, the simulation indicated the deepest scour at the final flow rate of *Q*_*f*_ = 2 *l/s*, with a depth of approximately 0.098 m. This comparison reveals a disparity in the rate of change, amounting to about 18.4%, between the experimental and numerical results.

Fig 4(B) displays the scour profile resulting from a 15% blockage at the inlet. Throughout the various stages of the hydrograph, specifically at the initial first three flow rates of *Q*_*r*_ = 2, 5, and 8 *l/s*, the observed data’s scour profiles and those from the numerical analysis follow one another sequentially. However, as the hydrograph progresses beyond these initial rates, the pattern of change continues for the numerical data, whereas in the experimental observations, the outcomes of subsequent stages diverge significantly from the first three flow rates. The deepest scour occurred at an increasing flow rate of *Q*_*r*_ = 11 *l/s*, reaching almost 0.108 m, whereas, in the numerical analysis, the greatest scour depth was observed at the final flow rate of *Q*_*f*_ = 2 *l/s*, measuring approximately 0.114 m. This demonstrates that the maximum scour depth in the numerical data exceeded that observed in the experimental data by 5.26%.

Fig 4(C) presents the scour profile for a 30% inlet obstruction. In this scenario, the scouring pattern is similar to the one observed with a 15% blockage, particularly at the onset of the hydrograph for flow rates *Q*_*r*_ = 2 and *l/s*, where the numerical and observed data are closely aligned. Beyond these initial rates, the experimental data demonstrates a marked deviation from the first couple of stages, a contrast not evident in the numerical data. Here, the numerical data consistently reflects an increment in scour depth proportional to the flow rate, without significant deviation between stages. In instances of a 30% inlet blockage, the greatest scour depth recorded in the experimental setup occurred at a flow rate of *Q*_*f*_ = 5 *l/s*, reaching approximately 0.135 m, whereas the numerical simulations showed the maximum scour depth at a flow rate of *Q*_*f*_ = 2 *l/s*, reaching approximately 0.126 m. This comparison reveals that the experimental data’s maximum scour depth exceeded that of the numerical data by 6.7%.

The comparative analysis of the observed and numerical data showed that in scenarios with no blockage and with a 15% blockage, the numerical measurements of maximum scour depth exceeded those from the experimental data. Conversely, with a 30% blockage, the experimental data revealed a greater maximum scour depth compared to the numerical data.

Further analysis highlighted the significant impact of a 30% blockage, as observed in the second hydrograph, on the development of scour profiles. This effect was more pronounced than in unblocked or 15% blocked conditions. In numerical assessments, the depth of scour for a 30% blockage exceeded those of no blockage and a 15% blockage by 22% and 9.5%, respectively. Moreover, experimental observations recorded the deepest scour holes in the 30% blockage scenario, surpassing those in unblocked and 15% blocked conditions by 41% and 20%, respectively [9, 10].

Table 2 provide a detailed evaluation of the upstream (*h*_*u*_) and downstream flow depths (*h*_*d*_), Froude Number (*F*_*r*_), Densimetric Froude Number (*F*_*rd*_), critical flow velocity (*V*_*c*_*)* and the maximum scour depth (*d*_*sm*_) and its location (*X*_*sm*_) for the culvert under the first and second hydrograph’s unsteady flow conditions.

**Table 2:** Numerical tests condition for unsteady conditions.

Blockage (%)	*Q* (*l/s*)	*t* (min)	*h*_*u*_ *(mm)*	*h*_*d*_ *(mm)*	*F*_*r*.*u*_	*F*_*r*.*d*_	*F*_*rd*.*u*_	*F*_*rd*.*d*_	*V*_*c*. *U*_ *(m/s)*	*V*_*c*. *d*_ *(m/s)*	*d*_*sm*_ *(mm)*	*X*_*sm*_ *(mm)*
	*Q*_*m*_ = 22 *l/s*											
0	6	40	8.5	3.52	0.129	0.483	1.719	4.152	0.244544	0.187128	62.47	120
	10	80	12	5.4	0.127	0.419	2.024	4.478	0.267002	0.214998	73	240
	14	120	14.3	7.45	0.138	0.367	2.387	4.577	0.278422	0.235957	78.5	280
	18	160	16.3	6.58	0.146	0.567	2.689	6.662	0.286947	0.227869	89	280
	22	200	18	8.44	0.153	0.477	2.973	6.348	0.293408	0.244082	96.3	320
	18	240	16.1	6.94	0.149	0.524	2.727	6.317	0.286143	0.231338	101	360
	14	280	14.42	7.14	0.136	0.390	2.364	4.775	0.278966	0.233189	103.5	360
	10	320	12.23	5.3	0.124	0.436	1.991	4.595	0.268238	0.213781	107	300
	6	360	11.1	4.12	0.086	0.382	1.316	3.547	0.261924	0.197379	109	300
15	6	40	8.721	0.95	0.124	3.35	1.68	15.38	0.246215	0.10183	64.2	140
	10	80	12.03	2.27	0.127	1.55	2.02	10.72	0.267164	0.158559	89.64	200
	14	120	15.48	3.94	0.122	0.95	2.20	8.65	0.283585	0.194469	94.6	240
	18	160	18.51	4.31	0.120	1.07	2.37	10.18	0.295227	0.200315	99.3	340
	22	200	21.78	5.04	0.115	1.04	2.46	10.62	0.305822	0.210505	106	320
	18	240	18.49	3.57	0.120	1.42	2.37	12.28	0.255969	0.188047	111	260
	14	280	17.36	3.42	0.103	1.18	1.96	9.97	0.29105	0.185251	116.2	260
	10	320	12.75	2.77	0.117	1.15	1.91	8.79	0.27095	0.171523	118	340
	6	360	10.13	2.43	0.099	0.843	1.44	6.01	0.255969	0.162995	118.4	340
30	6	40	9.02	3.27	0.12	0.54	1.62	4.47	0.248411	0.18233	73.3	160
	10	80	13.02	3.79	0.11	0.72	1.87	6.43	0.272314	0.191941	86.3	220
	14	120	14.84	4.29	0.13	0.84	2.29	7.94	0.280836	0.200012	99.6	180
	18	160	16.89	6.03	0.14	0.65	2.59	7.27	0.289262	0.222185	101.2	200
	22	200	18.36	6.01	0.15	0.80	2.92	8.92	0.294697	0.221968	110.2	260
	18	240	16.39	6.42	0.14	0.59	2.67	6.83	0.287305	0.226266	117	300
	14	280	15.18	6.04	0.13	0.50	2.25	5.64	0.282311	0.222293	121	340
	10	320	12.75	4.12	0.12	0.64	1.91	5.91	0.27095	0.197379	125.5	360
	6	360	11.13	3.42	0.09	0.50	1.31	4.27	0.2621	0.185251	126.8	360
	*Q*_*m*_ = 14 *l/s*											
0	2	25	4.91	1.48	0.29	1.77	2.98	9.88	0.208803	0.130702	42.8	100
	5	50	7.85	2.55	0.24	1.30	3.10	9.54	0.239363	0.166134	79.5	160
	8	75	10.18	2.93	0.23	1.48	3.35	11.64	0.25629	0.17518	82.25	120
	11	100	12.22	3.34	0.22	1.57	3.59	13.12	0.268185	0.18371	84.66	140
	14	125	14.18	8.62	0.22	0.46	3.78	6.22	0.277873	0.245457	89.8	280
	11	150	14.15	11.63	0.18	0.24	3.10	3.77	0.277735	0.264962	89.6	180
	8	175	13.88	9.13	0.14	0.27	2.46	3.73	0.27648	0.2492	95.9	220
	5	200	10.20	8.10	0.16	0.23	2.39	3.01	0.256417	0.241404	96.95	240
	2	225	9.31	6.42	0.11	0.20	1.57	2.28	0.250471	0.226266	98.4	220
15	2	25	4.41	1.65	0.34	1.51	3.31	8.88	0.201809	0.137783	38.9	140
	5	50	5.57	1.82	0.40	2.17	4.37	13.38	0.217017	0.14417	78.2	180
	8	75	5.56	3.09	0.57	1.37	6.13	11.03	0.2169	0.178643	92.8	180
	11	100	11.72	3.45	0.24	1.49	3.74	12.71	0.265464	0.18582	99.2	220
	14	125	12.45	4.82	0.26	1.10	4.30	11.11	0.269399	0.207598	103.2	240
	11	150	13.61	5.04	0.19	0.84	3.22	8.70	0.275201	0.210505	106.4	280
	8	175	12.97	4.91	0.16	0.68	2.63	6.94	0.272064	0.208803	104.6	300
	5	200	10.63	3.92	0.15	0.68	2.29	6.21	0.259107	0.194138	111.6	300
	2	225	9.63	2.40	0.11	0.86	1.52	6.09	0.252672	0.162186	113.7	320
30	2	25	6.04	3.42	0.21	0.50	2.42	4.27	0.222293	0.185251	37.5	120
	5	50	8.91	4.11	0.20	0.64	2.73	5.92	0.247611	0.19722	70.8	140
	8	75	9.82	6.93	0.24	0.408	3.47	4.92	0.253945	0.231244	104.3	260
	11	100	13.43	8.14	0.19	0.41	3.26	5.38	0.274334	0.241725	111.4	280
	14	125	17.43	10.1	0.19	0.36	3.07	5.30	0.291312	0.255776	116.6	300
	11	150	19.31	12.71	0.11	0.21	2.27	3.44	0.297983	0.270745	119.7	300
	8	175	18.91	14.13	0.09	0.14	1.80	2.41	0.29662	0.277643	121.8	340
	5	200	16.31	12.12	0.08	0.13	1.49	2.01	0.286987	0.26765	123.9	360
	2	225	15.69	11.13	0.05	0.08	0.93	1.31	0.284463	0.2621	125.96	380

The data show that downstream measurements of *F*_*r*_ and *F*_*rd*_ are consistently higher than upstream, regardless of whether the culvert is non-blocked, a 15% blockage, or a 30% blockage. This indicates a significant enhancement in hydraulic activity after the water passes through the culvert, a trend that remains constant across all blockage conditions, reflecting stable and increased fluid dynamics downstream.

The analysis of critical flow velocities for different discharge rates reveals important insights. For a maximum discharge *Q*_*m*_ = 22 *l/s*, the critical velocities ranged from 0.24 m/s to 0.30 m/s upstream and from 0.10 m/s to 0.24 m/s in downstream. In comparison, for a *Q*_*m*_ = 14 *l/s*, the critical velocities were between 0.20 m/s and 0.29 m/s upstream and between 0.13 m/s and 0.27 m/s downstream. These results indicate that higher discharge rates result in higher critical velocities upstream, suggesting a reduction in flow energy downstream. For lower discharge rates, the critical velocities are more consistent between upstream and downstream locations. This pattern underscores the need to consider both discharge rates and flow depths when evaluating critical flow velocities to understand their impact on scour processes around culverts, ultimately aiding in the design of more stable hydraulic structures.

### 3.2 Steady flow conditions

**Fig 5:**
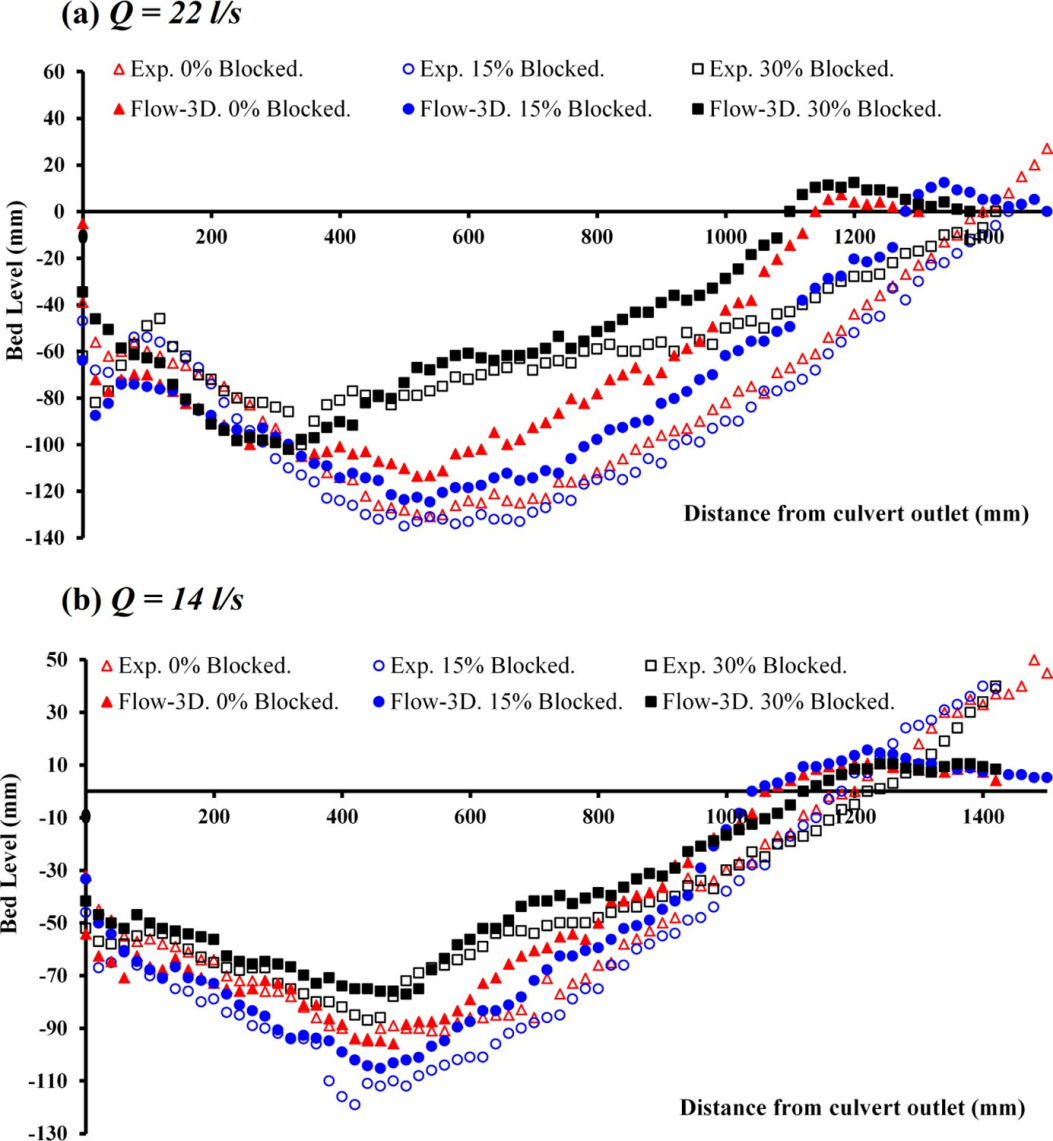
Scour profile for steady conditions: a) *Q* = 22 *l/s*, *t* = 360 min, and b) *Q* = 14 *l/s*, *t* = 225 min.

Fig 5(A) illustrates a comparison of the physical and numerical data on scour profiles at a flow rate of *Q*_*m*_ = 22 *l/s* under inlet blockages of 0%, 15%, and 30% for *t* = 360 min. The observed data for an unobstructed flow showed a scour depth maximum (*d*_*sm*_) of approximately 0.131 m, increasing to almost 0.135 m for a 15% blockage, and decreasing to nearly 0.1 m for a 30% blockage. The numerical data for these blockage conditions of 0, 15, and 30% recorded *d*_*sm*_ values of approximately 0.114, 0.125, and 0.102 m, respectively. The comparison of discrepancies in *d*_*sm*_ (scour depth maximum) between physical observations and numerical simulations demonstrated that for scenarios without blockage and with a 15% obstruction, the observed values were higher by 13% and 7.4%, respectively, in contrast to the numerical findings. In the case involving a 30% blockage, numerical data surpassed physical measurements by 2%.

This analysis clarifies that for both the observed and simulated datasets, the scenario with a 15% blockage leads to the most significant scour depth maximum (*d*_*sm*_), followed by the 30% blockage and the unobstructed case. Hence, it becomes clear that an increase in blockage percentage does not linearly render an increase in scour depth. Additionally, the configuration with a 15% blockage produced the largest scour hole downstream, followed by the unblocked scenario ranking second and the 30% blockage scenario having the least impact.

Fig 5(B) depicts an analysis comparing the development of scour profiles from both observed and numerical data at the downstream positions of a circular culvert under steady flow conditions. In Fig 5(B), the analysis focuses on the variation between physical and numerical data concerning the formation of scour at a second-tier flow rate of *Q*_*m*_ = 14 *l/s*, taking into account different degrees of blockage for *t* = 225 min. The data demonstrates that sediment displacement is discernible across all scenarios, regardless of whether the data is physical or numerical. When comparing physical and numerical data regarding the maximum depth of scour, in an unobstructed condition, the depths recorded were approximately 0.094 m for the experimental data and 0.096 m for the numerical data. With a 15% blockage, the observed depths were almost 0.119 m, and simulations showed 0.105 m. At 30% obstruction, physical measurements recorded approximately 0.087 m, while the numerical data indicated 0.077 m. When comparing the rate of difference between the physical and numerical data for *d*_*sm*_, it was determined that for the unblocked condition, numerical results were higher by 2.1%. Conversely, for the 15% and 30% blockage scenarios, the observed data recorded higher values by 11.8% and 11.5% compared to the numerical data, respectively. The findings demonstrated that the escalation in blockage percentage does not directly correlate with the size of scour holes forming downstream. Contrary to expectations, the culvert experiencing a 15% blockage developed the largest scour hole, followed by the unobstructed scenario and the 30% obstruction case.

The present research highlights significant patterns under conditions of constant flow. When the flow rate was adjusted from 14 *l/s* to 22 *l/s*, there was a noticeable increase in scour depth downstream of the culvert. However, an escalation in blockage did not uniformly result in a greater depth of scour across all instances. Instead, fluctuations occurred with certain scenarios exhibiting deeper scour depths. This variation was likely due to water spilling over the blockage barrier, particularly at higher levels of obstruction. As a result, in such instances, the velocity of the water was reduced in comparison to other scenarios, affecting the scouring dynamics [7, 9, 10].

Table 3 presents the same evaluated parameters as detailed in the previous section but for the steady-state cases of *Q*_*m*_ = 22 l/s and *Q*_*m*_ = 14 l/s. The data shown that the *F*_*r*_ and *F*_*rd*_ for downstream flow are greater than upstream flow because the Froude number downstream is often higher than upstream due to changes in flow conditions induced by the culvert. The culvert constricts the flow, which increases the flow velocity as water exits the culvert due to the continuity equation. This increased velocity, coupled with a reduction in flow depth, results in a higher Froude number downstream.

**Table 3:** Numerical tests condition for unsteady case.

Steady Case	Blockage (%)	*Q* (*l/s*)	*t* (min)	*h*_*u*_ *(mm)*	*h*_*d*_ *(mm)*	*F*_*r*.*u*_	*F*_*r*.*d*_	*F*_*rd*.*u*_	*F*_*rd*.*d*_	*V*_*c*. *U*_ *(m/s)*	*V*_*c*. *d*_ *(m/s)*	*d*_*sm*_ *(mm)*	*X*_*sm*_ *(mm)*
***Q***_***m***_ **= 22 *l/s***	0	22	360	16.7	9.39	0.17	0.41	3.20	5.71	0.288526	0.251029	113.5	520
	15		360	19.61	5.98	0.135	0.80	2.73	8.95	0.298987	0.221642	124.6	540
	30		360	22.68	6.98	0.108	0.635	2.36	7.68	0.308459	0.231713	102	320
***Q***_***m***_ **= 14 *l/s***	0	14	225	14.16	8.39	0.22	0.48	3.78	6.38	0.277781	0.243695	95.86	480
	15		225	17.95	4.80	0.15	1.11	2.98	11.15	0.293227	0.207327	105.2	460
	30		225	21.26	4.39	0.12	1.27	2.52	12.2	0.304248	0.201513	77.11	500

In analyzing critical flow velocities for different discharge rates, it is consistently observed that upstream velocities are higher compared to downstream. The velocities upstream range between 0.27 m/s to 0.31 m/s, while downstream velocities range between 0.20 m/s and 0.25 m/s.

### 3.3 Maximum scour depth

#### 3.3.1 First hydrograph

The depth and location of the scour at the outlet of a culvert play a pivotal role in determining culvert design and safety [10].

**Fig 6:**
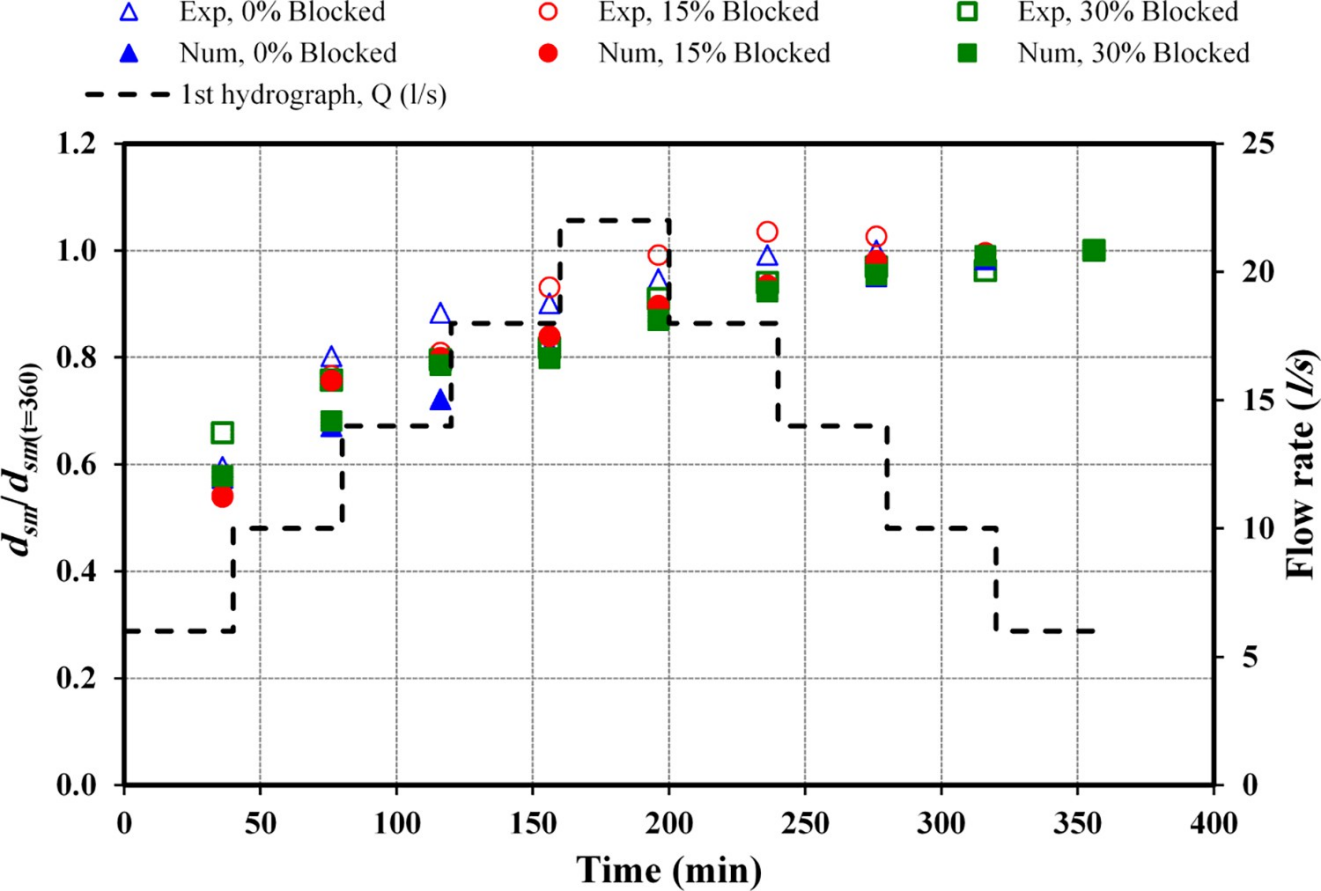
Comparison of *d*_*sm*_ between observed and numerical data (*Q*_*m*_ = 22 *l/s*).

Fig 6 offers a comparison of the deepest scour depths recorded in both physical and numerical studies within circular culvert formations under unsteady flow conditions for the first and second hydrographs at different rates of blockage. To standardize the maximum scour depth, the *d*_*s*_/*d*_*sm*_ ratio was utilized, where *d*_*s*_ signify the scour depth and *d*_*sm*_ represents the highest scour depth observed. The peak *d*_*sm*_ is noted at t = 360 minutes, starting with an initial flow rate from *Q*_*r*_ = 6 *l/s* to *Q*_*m*_ = 22 *l/s*, before decreasing back to *Q*_*f*_ = 6 *l/s* for the first hydrograph. For the second hydrograph, the flow begins at *Q*_*r*_ = 2 *l/s*, peaks at *Q*_*m*_ = 14 *l/s*, and then decreases to *Q*_*f*_ = 2 *l/s*.

In Fig 6, regarding the first hydrograph with *Q*_*m*_ = 22 *l/s*, in the case of an unblocked inlet culvert, the variation rates between physical and numerical data across the entire flow range, from the initial rise at *Q*_*r*_ = 6 *l/s* to the peak *Q*_*m*_ = 22 *l/s*, and the decrease to *Q*_*f*_ = 6 *l/s* were recorded as 5.3, 18, 20, 11, 8.3, 8.2, 6.8, 2.8 and 2.0%, respectively. This pattern indicates that in every scenario, the *d*_*sm*_ values obtained from physical measurements exceeded those recorded in the numerical data.

With a 15% blockage at the inlet, the analysis of the *d*_*sm*_ rate differences between the physical and numerical datasets for a 15% blockage revealed that in the initial three ascending steps at flow rates of 6, 10, and 14 *l/s*, numerical data exceeded physical data by margins of 3.5%, 1.9%, and 1.7%, respectively; and for the latter two descending steps at 10 and 6 *l/s*, the numerical data exceeded the physical data by margins of 3.3% and 2.9%. In contrast, physical *d*_*sm*_ values surpassed numerical ones at flow rates of *Q*_*r*_ = 18 *l/s*, *Q*_*m*_ = 22 *l/s*, and *Q*_*f*_ = 18 and 14 *l/s* by 7.2%, 6.9%, 7%, and 1.5%, respectively.

For a 30% inlet obstruction, both observed and calculated data demonstrated a steady escalation in *d*_*sm*_. The rate of change in *d*_*sm*_ between the physical and numerical data revealed that for all flow rates, the physical data results surpass those from the numerical analyses. During the increase in flow rates at 6, 10, 14, 18 *l/s* up to the maximum of 22 *l/s*, the variation percentages were approximately 15.8, 13.7, 5.2, 6.3, and 8.1%, respectively. This trend of differential rates continued into the decreasing flow phases of 18, 14, 10, and 6 *l/s*, with respective discrepancies of approximately 5.7, 5.5, 1.2%, and 4%.

To summarize, the collective findings from both the physical and numerical data demonstrate that heightened inlet blockage corresponds to increased *d*_*sm*_ values. The condition of a 15% blockage yielded deeper scour depths than those observed in unobstructed scenarios, and a 30% blockage condition further amplified scour depths beyond both the unblocked and 15% blocked scenarios. These outcomes highlight the complex interplay between flow rate and blockage degree in determining scour formation [9, 10].

#### 3.3.2 Second hydrograph

**Fig 7:**
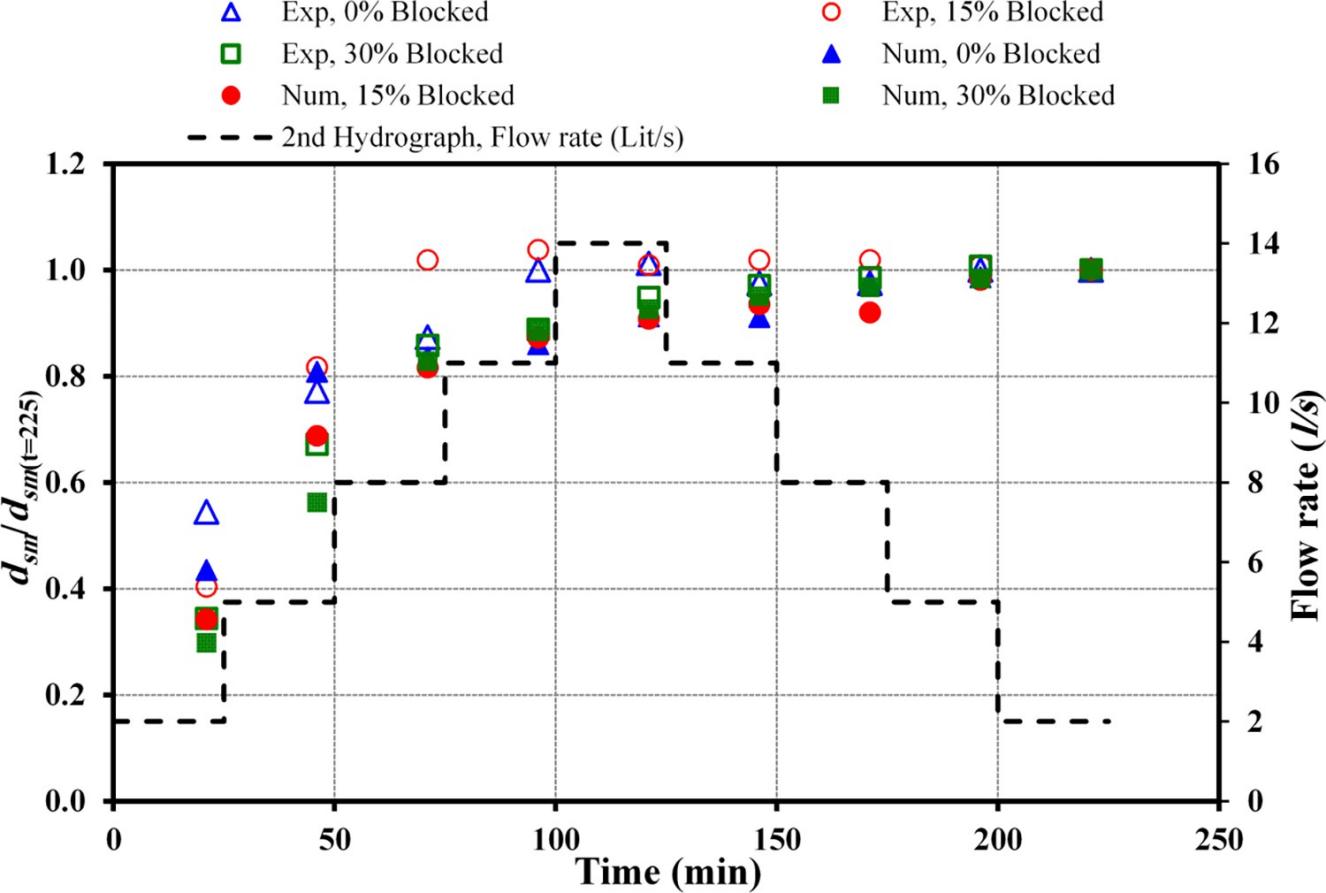
Comparison of *d*_*sm*_ between observed and numerical data (*Q*_*m*_ = 14 *l/s*).

Fig 7 illustrates the maximum scour depth (*d*_*sm*_) from both physical and numerical data against varying blockage intensities captured at t = 225 minutes. This encompasses a flow rate beginning at *Q*_*r*_ = 2 *l/s*, peaking at *Q*_*m*_ = 14 *l/s*, and subsequently diminishing to *Q*_*f*_ = 2 *l/s* within the framework of the second hydrograph.

In the scenario of a circular culvert analysed through an identical stepwise hydrograph, escalating inlet blockage rates invariably resulted in increased scour depths. For instance, in scenarios without blockage, the analysis comparing physical to numerical data revealed that across all stages of the hydrograph, the numerical maximum scour depth (*d*_*sm*_) values exceed those of the physical data. The variance in percentages for the ascending phases from *Q*_*f*_ = 2 *l/s* to *Q*_*m*_ = 14 *l/s* is approximately 0.4, 23, 16, 6.7, and 10.9%. This trend of increase persists through the subsequent phases of the hydrograph, concluding at *Q*_*f*_ = 2 *l/s* with differences noted at 14.1, 19.8, 18.5, and 19.7%.

With a 15% blockage at the inlet of the culvert, in the comparative analysis of physical versus numerical datasets across equivalent progressive stages for flow rates transitioning from *Q*_*r*_ = 6 *l/s* to *Q*_*m*_ = 14 *l/s* and then to *Q*_*f*_ = 8 *l/s*, the physical dataset exhibited a higher incidence of *d*_*sm*_ than the numerical dataset, with differences in percentages noted at 7.4, 8.0, 12.4, 1.7, 0.4, and 1.3%, respectively. Conversely, for the subsequent flow rates, the numerical dataset demonstrated greater *d*_*sm*_ accuracy than the physical data, particularly at *Q*_*f*_ = 5 and 2 *l/s*, with variances amounting to 5.9% and 8.5%, respectively.

When evaluating the impact of a 30% obstruction at the inlet, the comparison of *d*_*sm*_ percentages between actual observations and modelled data shows that *d*_*sm*_ events were more frequently recorded in the experimental data across all incremental stages of the hydrograph. Evaluating the changes from *Q*_*r*_ to *Q*_*m*_ and then to *Q*_*f*_, the differences were noted at 18.5%, 21.3%, 9.3%, 8.2%, 7.9%, 7.7%, 8.2%, and 6%, respectively, with the greatest discrepancy observed when the flow rate was at *Q*_*r*_ = 5 *l/s*.

In conclusion, the aggregated data from both the experimental and computational analyses reveal a direct correlation between increased inlet obstruction and elevated *d*_*sm*_ values. Scenarios with a 15% inlet blockage resulted in greater scour depths compared to conditions without any blockage. Furthermore, a blockage of 30% intensified the scour depths even more, surpassing both the unobstructed and 15% obstruction cases. During the *d*_*sm*_ analysis comparing experimental to computational data, it was found that under no blockage conditions, the computational data was higher. However, with a 15% obstruction, the experimental data surpassed the computational data, except at the final two stages of the hydrograph. Moreover, under a 30% obstruction, the experimental data exceeded the computational findings [9, 10].

### 3.4 Maximum scour depth location

**Fig 8:**
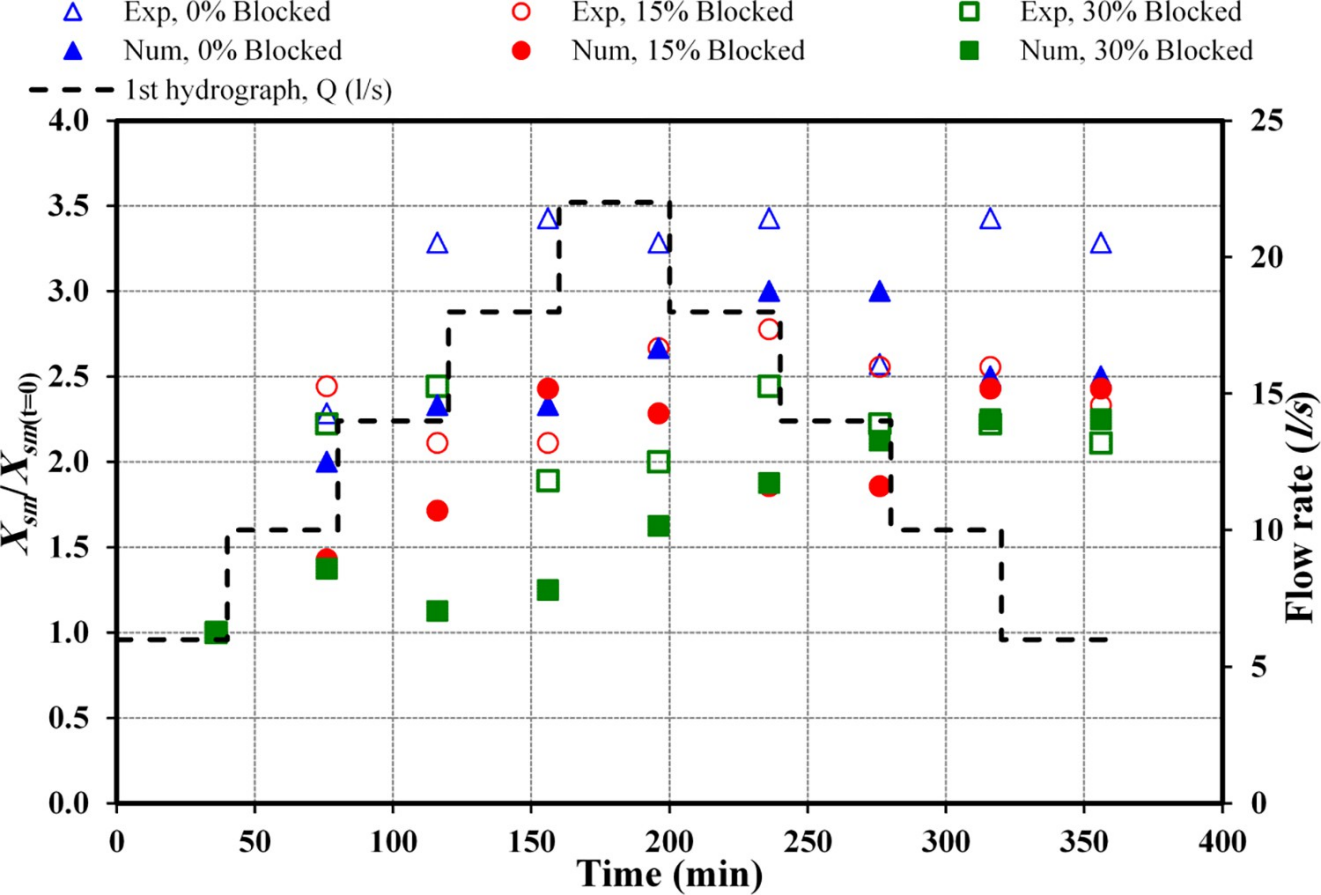
Comparison of *X*_*sm*_ between observed and numerical data (*Q*_*m*_ = 22 *l/s*).

**Fig 9:**
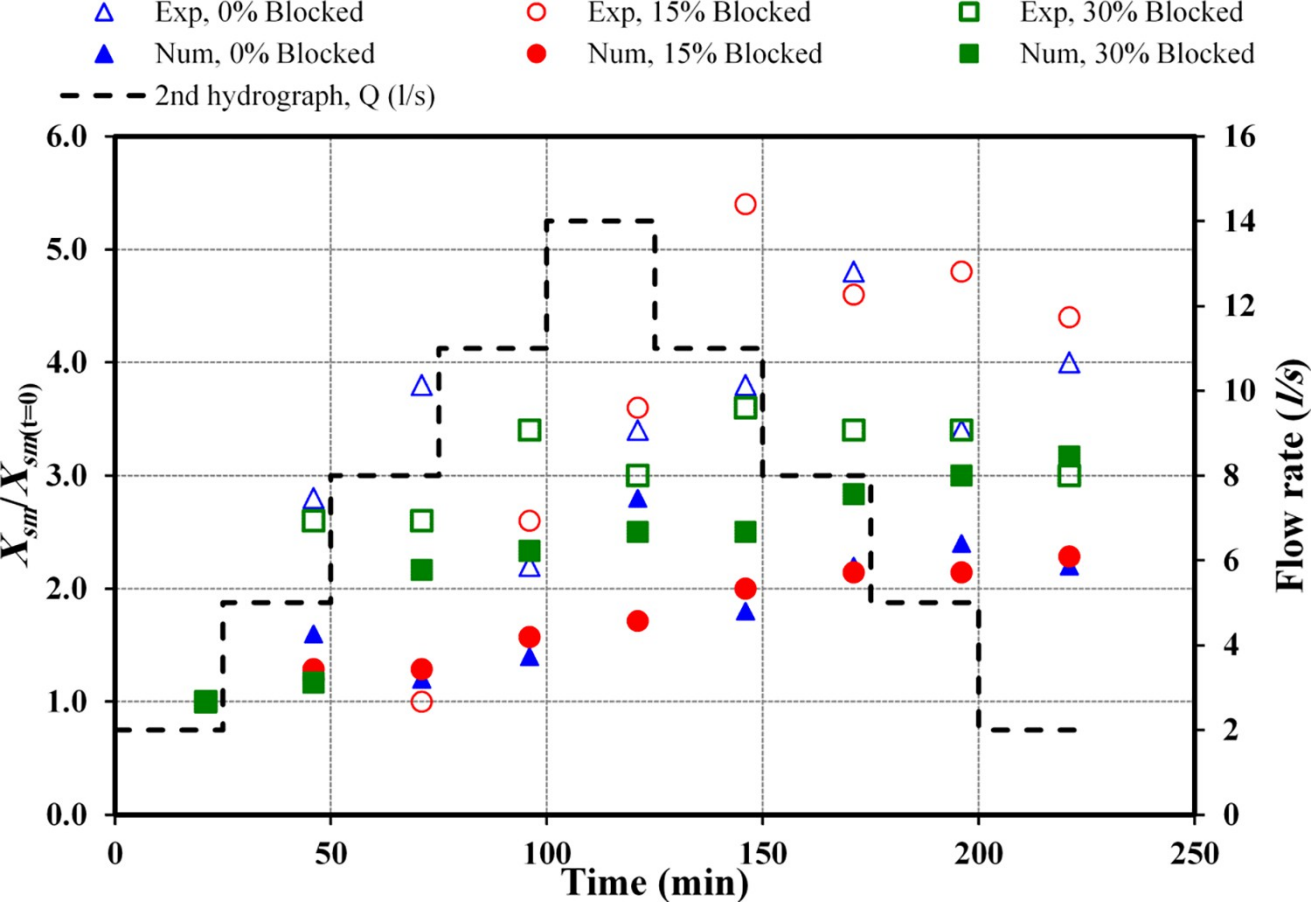
Comparison of *X*_*sm*_ between observed and numerical data (*Q*_*m*_ = 14 *l/s*).

Figs 8 and 9 highlight the exact locations of the deepest scouring observed in both the physical and numerical models, accounting for different blockage intensities and drawing on data from both the initial and follow-up hydrographs. To identify the deepest scour locations, the study employed the *X*_*s*_/*X*_*sm*_ ratio, where *X*_*s*_ indicates the horizontal distance from the culvert’s outlet and *X*_*sm*_ pinpoints the location of the deepest scour at multiple time points, starting from the initial position at *t* = 0 minutes and taking into account the direction of water flow. These ratios offer critical insights into the dynamics of scouring within the culvert infrastructure.

Fig 8 delineates the analysis between directly measured and modelled data for the initial hydrograph. Investigating a circular culvert under a uniform obstruction level, this study aimed at locating the peak scour depths throughout various hydrograph stages. For scenarios without blockage, the evolution was divided into three distinct phases. Initially, the maximum scour depth (*X*_*sm*_) was pinpointed 0.14 m away from the inlet, later transitioning to 0.32 m and then to 0.46 m, before reaching its maximum extension of 0.48 m as the flow escalated to *Q*_*r*_ = 18 *l/s*. With the flow at *Q*_*m*_ = 22 *l/s*, *X*_*sm*_ settled at 0.46 m. During the final flow phase (*Q*_*f*_), slight variations in these positions were observed and listed as 0.48 m, 0.36 m, 0.48 m, and 0.46 m distant from the outlet. Conversely, under identical obstruction and hydrograph phase conditions in the simulation models, the maximum scour depth (*X*_*sm*_) was positioned closer to the culvert’s outlet. Initially, *X*_*sm*_ was found at 0.12 m from the inlet, moving to 0.24 m and then to 0.28 m; it remained at 0.28 m as the flow rate achieved *Q*_*r*_ = 18 *l/s*. At a flow rate of *Q*_*m*_ = 22 *l/s*, *X*_*sm*_ was located at 0.32 m. In the *Q*_*f*_ phase, the recorded positions showed minor changes, documented as 0.36 m, 0.36 m, 0.30 m, and 0.30 m from the outlet.

In scenarios involving a 15% obstruction within physical models, the *d*_*sm*_ displayed a series of changing positions, ultimately recording distances of 0.18 m, 0.44 m, 0.38 m, 0.38 m, and 0.48 m from the outlet as the flow transitioned from *Q*_*r*_ to *Q*_*m*_. At the commencement of the *Q*_*f*_ phase, the *d*_*sm*_ was observed at its greatest distance, 0.50 m, from the outlet. For the subsequent flow rates, its positions were noted to be 0.46 m, 0.46 m, and 0.42 m away from the inlet. Regarding the numerical model, the initial four flow rates witnessed the *d*_*sm*_ achieving its utmost *X*_*s*_ from the outlet at distances of 0.14 m, 0.20 m, 0.24 m, and 0.034 m. In contrast, for the following two flow rates, specifically at *Q*_*m*_ = 22 *l/s* and *Q*_*f*_ = 18 *l/s*, the *X*_*sm*_ was observed to be marginally closer to the outlet, at approximately 0.26 m for both instances. Yet, in the final two stages, the maximum *X*_*sm*_ extended to 0.34 m.

In the context of a 30% obstruction, experimental observations revealed variability in *d*_*sm*_ locations. Throughout the initial increase phase, the *d*_*sm*_ was identified at distances roughly estimated at 0.18 m, 0.40 m, 0.44 m, 0.34 m, and 0.36 m. During the decline phase, the *d*_*sm*_ positions were recorded at approximately 0.44 m, 0.40 m, 0.40 m, and 0.38 m from the outlet. In the case of the numerical simulations, a similar pattern of fluctuation was noted where DSM positions during the increase phase up to the peak flow rate were marked at about 0.16 m, 0.22 m, 0.18 m, 0.20 m, and 0.26 m. For the decline phase, variations in *d*_*sm*_ were observed, peaking in the last two increments at approximately 0.30 m, 0.34 m, 0.36 m, and 0.36 m.

In conclusion, the collective analysis reveals that when experimental and model-based data with equivalent obstruction are compared, the maximal scour depth location is often observed to be more distant from the outlet in the experimental setups.

Fig 9 demonstrates the impact of changes in the second hydrograph on the deepest point of scouring, *X*_*sm*_, across both the observed and computational datasets at varying levels of obstruction. In a scenario where the box culvert remains unobstructed, *X*_*sm*_ is initially found to be approximately 0.10 m away from the outlet. As the flow rates increase (5, 8, 11, up to *Q*_*m*_ = 14 *l/s*), *X*_*sm*_ moves to locations such as 0.28, 0.38, 0.22, and finally 0.34 m in the order mentioned. Throughout the *Q*_*f*_ stage, the positions are observed at 0.38, 0.48, 0.34, and 0.4 m, respectively, with the most distant downstream location of *d*_*sm*_ recorded at *Q*_*f*_ = 5 *l/s*. In contrast, within the numerical models, *X*_*sm*_ grows in a manner directly correlated to the flow rates, marked at 0.10, 0.16, 0.12, and 0.14 m, with the highest *X*_*sm*_ forecasted to be around 0.28 m at *Q*_*m*_ = 22 *l/s*. During the declining phase, the outcomes show variability, being documented at 0.18, 0.22, 0.24, and 0.22 m.

In scenarios with a 15% blockage, the *d*_*sm*_ observed in the physical model demonstrated varied locations, reaching distances of 0.10, 0.12, 0.10, 0.26, and 0.36 m from the inlet from *Q*_*r*_ to *Q*_*m*_. At the beginning of the *Q*_*f*_ phase, the *d*_*sm*_ was located at its farthest point, 0.54 m from the outlet. For the subsequent flow rates, it was positioned at 0.46, 0.48, and 0.44 m from the inlet. However, in the numerical model, the maximum scour depth (*X*_*sm*_) gradually moved further away from the culvert outlet through each phase of the hydrograph. From *Q*_*r*_ to *Q*_*m*_, the distances were recorded as approximately 0.14, 0.18, 0.18, 0.22, and 0.24 m, and for the final *Q*_*f*_ phase, the distances were almost 0.28, 0.30, 0.30, and 0.32 m. Thus, the greatest *d*_*sm*_ was observed with the last *Q*_*f*_ = 2 *l/s*.

In the case of a 30% obstruction, the *d*_*sm*_’s location in the observed dataset demonstrated variability. Throughout the initial increase, the recorded locations ranged from 0.10 m, 0.26 m, 0.26 m, 0.34 m, to 0.30 m. The peak *X*_*sm*_ was observed at the first *Q*_*f*_ = 18 *l/s*, reaching 0.36 m, while during the subsequent decline, the positions settled around 0.34 m, 0.34 m, and 0.30 m from the outlet. However, in the simulation data, as each phase progressed, the *X*_*sm*_ moved increasingly further from the outlet. Starting from the initial *Q*_*r*_ to *Q*_*m*_ and then to *Q*_*f*_, the *X*_*sm*_ sequence was 0.12, 0.14, 0.26, 0.30, 0.30, 0.34, 0.36, and 0.38 m, respectively.

In summary, the collective results demonstrated that the *d*_*sm*_ points, as determined from experimental observations, are generally positioned at greater distances from the culvert’s outlet when compared to their counterparts in numerical models. This finding consistently illustrates a notable difference in the spatial occurrence of maximum scour depth (*X*_*sm*_) between the physical experiments and simulated data, emphasizing the variation in proximity to the outlet.

### 3.5 Error measurement

**Fig 10:**
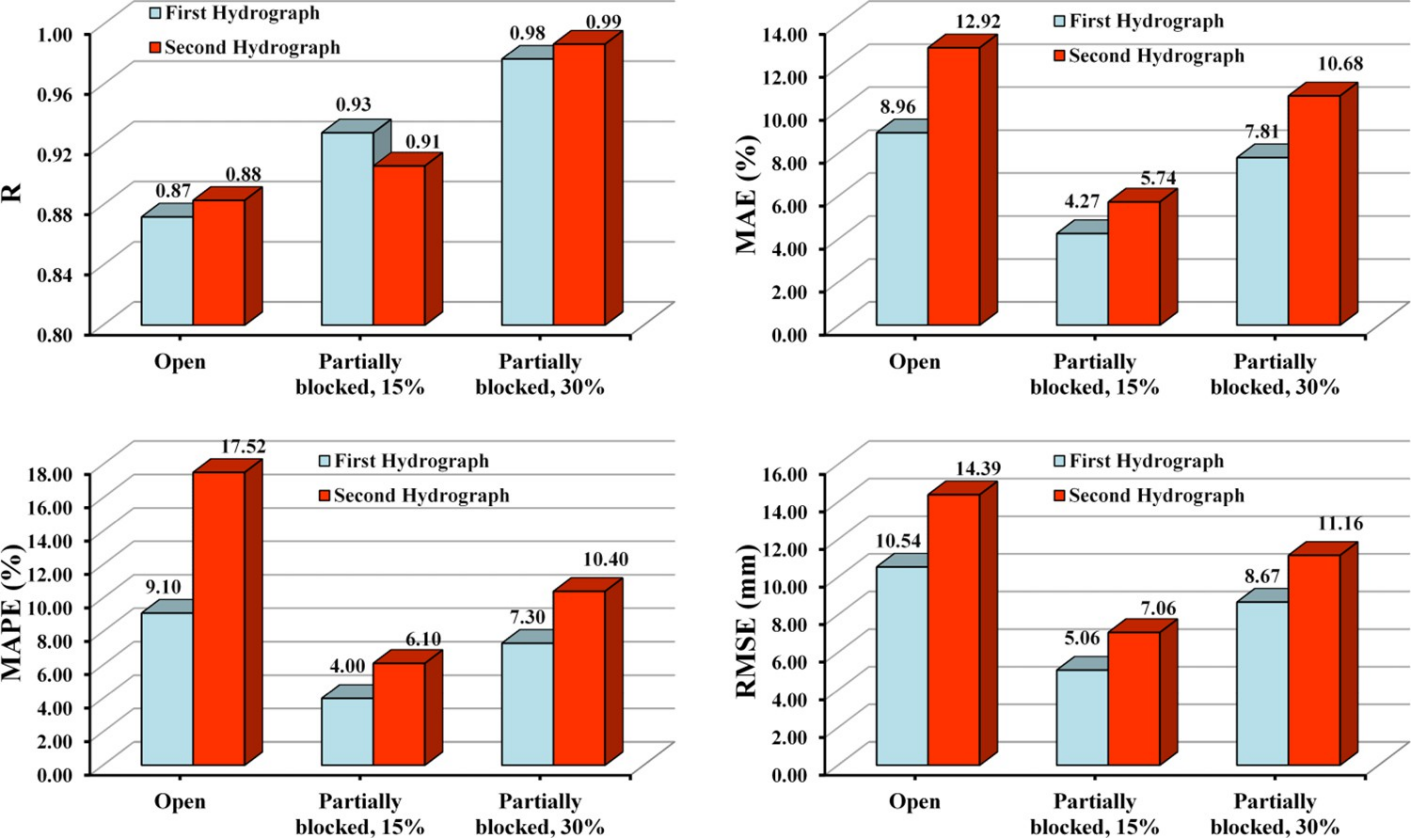
Statistical indicators of maximum scour depth evaluation of the experimental and numerical models.

The evaluation of the accuracy and correctness of the numerical simulation was compared with the laboratory data based on the four evaluation indicators used, separately, by the percentage of blockage and the studied hydrograph. The results showed that the MAE (%) for estimating the maximum scour depth in numerical simulation relative to observed data and for 0%, 15%, and 30% Blockage was 8.96, 4.27, and 7.81%, respectively, for the first hydrograph. Also, these values for the second hydrograph were 12.92, 5.74, and 10.68%, respectively. The process of changes in the values of evaluation indicators is presented in Fig 10. The statistical indicators indicated that the numerical model performs reasonably well predicting scour depth at the culvert outlet under different culvert blockage conditions and flow scenarios. The model demonstrates good correlation, low error values, and accurate predictions compared to the observed data, although there are variations in performance based on the specific conditions being evaluated.

## 4 Conclusion

In conclusion, the present study demonstrates the effectiveness of Flow-3D software in accurately predicting scour depth and location in circular culverts under various flow conditions. Through a comparison with experimental data, the research validates the software’s turbulence models, confirming its reliability for hydraulic engineering applications. The result findings are outlined below:

- Across both hydrographs, experimental results demonstrated more significant scour effects than numerical models, particularly with a 30% blockage, which heightened maximum scour depth by up to 14.2% over free-flow scenarios, signaling a rapid scour increase. The 30% blockage impact was profound, with numerical studies indicating a 22% deeper scour compared to unobstructed cases and experimental findings showing a 41% deeper scour relative to unblocked conditions. These observations underscore the substantial influence of a 30% blockage on scour depth intensification.
- Analysis under steady flow conditions indicated that a 15% blockage results in the deepest scour, challenging the notion that higher blockage levels lead to increased scour depths. The sequence of impact started with the 15% blockage causing the largest scour hole, while the 30% blockage had the least effect. Even with flow rates increased from 14 *l/s* to 22 *l/s*, higher blockages did not uniformly deepen scour, as overflow from obstructions can lower water speed and modify scour behavior.
- Across unsteady flow scenarios, data showed that greater blockage at the inlet consistently results in deeper scour depths. A 15% blockage significantly increased scour compared to the nonblocked, while a 30% blockage further enhanced scour depth, surpassing both unobstructed and 15% blockage effects. This indicates a direct link between blockage level and scour depth, where a 30% blockage resulted in the most pronounced increase.
- The collective results highlighted that the *d*_*sm*_ points, as determined from experimental observations, were generally positioned at greater distances from the culvert’s outlet when compared to their counterparts in numerical models. This finding consistently illustrates a notable difference in the spatial occurrence of maximum scour depth (*X*_*sm*_) between the physical experiments and simulated data, emphasizing the variation in proximity to the outlet.

The primary limitations of the numerical study on the scour process downstream of a circular culvert include the lack of comprehensive data, particularly high-resolution field data, which restricts the ability to fully validate the numerical model. The condition of the available data may not adequately represent real-world complexities, leading to potential inaccuracies in the model’s predictions. Additionally, the reliance on certain assumptions in the numerical modeling process, such as simplified flow conditions and uniform sediment properties, further limits the accuracy and generalizability of the results across different scenarios.

## Supporting information

S1 File The data set used in this research, in steady and unsteady flow conditions. (PDF)

## Abbreviations

d_50_: average sediments grain size
σ_g_: geometric standard deviation of sediment
$u_{{*}_{c}}$: critical shear velocity
V_c_: critical flow velocity
V: average of flow velocity
y_w_: water depth
R_e_: Reynolds number
Q: flow rate
Q_r_: rising flow rate in hydrographs
Q_m_: maximum flow rate
Q_f_: falling flow rate
W: width of the upstream channel
S_0_: bed slope
D: diameter of culvert
T: time
*ρ*: fluid density
*μ*: dynamic viscosity of water
*g*: acceleration due to gravity
H_e_: the flow depth at the culvert inlet
H_u_: the upstream flow depth
h_c_: culvert width
L_c_: culvert length
X_s_: horizontal distance from the culvert’s outlet
X_sm_: *=* pinpoints the location of the deepest scour
X*: non-dimensional blockage height
A_c_: total area of the culvert opening
B: rate of blockage
*F*_*re*_: Froude number at the culvert entrance
*F*_*ru*_: upstream flow Froude number
d_s_: actual scour depth
d_sm_: the maximum scour depth

## References

[1] Sorourian S., Keshavarzi A., Ball J., & Samali B. (2014). Blockage effects on scouring downstream of box culverts under unsteady flow. *Australasian Journal of Water Resources*, 18(2), 180–190.

[2] Othman Ahmed K., Kavianpour M. R., Amini A., & Aminpour Y. (2024). A state-of-the-art review of physical modeling of scouring at the culvert outlet. *ISH Journal of Hydraulic Engineering*, 1–8.

[3] Liriano S. L., Day R. A., & Rodney White W. (2002). Scour at culvert outlets as influenced by the turbulent flow structure. *Journal of Hydraulic Research*, 40(3), 367–376.

[4] Rigby E. H., Boyd M. J., Roso S., Silveri P., & Davis A. (2002). Causes and effects of culvert blockage during large storms. In *Global solutions for urban drainage* (pp. 1–16). doi: 10.1061/40644(2002)298

[5] Ho H. C., Muste M., & Ettema R. (2013). Sediment self-cleaning multi-box culverts. *Journal of hydraulic research*, 51(1), 92–101. doi: 10.1080/00221686.2012.757565

[6] Tan S. M., Lim S. Y., Wei M., & Cheng N. S. (2020). Application of particle densimetric Froude number for evaluating the maximum culvert scour depth. *Journal of Irrigation and Drainage Engineering*, 146(8), 04020020.

[7] Galán Á., & González J. (2020). Effects of shape, inlet blockage and wing walls on local scour at the outlet of non-submerged culverts: undermining of the embankment. *Environmental Earth Sciences*, 79(1), 25.

[8] Taha N., El-Feky M. M., El-Saiad A. A., & Fathy I. (2020). Numerical investigation of scour characteristics downstream of blocked culverts. *Alexandria Engineering Journal*, 59(5), 3503–3513.

[9] Günal M., Günal A. Y., & Osman K. (2019). Simulation of blockage effects on scouring downstream of box culverts under unsteady flow conditions. *International Journal of Environmental Science and Technology*, 16, 5305–5310.

[10] Othman Ahmed K., Amini A., Bahrami J., Kavianpour M. R., & Hawez D. M. (2021). Numerical modeling of depth and location of scour at culvert outlets under unsteady flow conditions. *Journal of Pipeline Systems Engineering and Practice*, 12(4), 04021040.

[11] Ahmed K. O., Nariman N., Hawez D. M., Kisi O., & Amini A. (2023). Predicting and Optimizing the Influenced Parameters for Culvert Outlet Scouring Utilizing Coupled FLOW 3D-Surrogate Modeling. *Iranian Journal of Science and Technology*, *Transactions of Civil Engineering*, 47(3), 1763–1776.

[12] Ahmed K. O., Kavianpour M. R., Amini A., & Aminpour Y. (2024). Physical modeling of the effect of shape, blockage, and flow variability on scour in culvert outlets. *Plos one*, 19(6), e0306252. doi: 10.1371/journal.pone.0306252 38935745 PMC11210847

[13] Kothe D. B., Mjolsness R. C., & Torrey M. D. (1991). *RIPPLE*: *A computer program for incompressible flows with free surfaces*. Available to DOE and DOE contractors from OSTI.

[14] Hirt C. W., & Nichols B. D. (1981). Volume of fluid (VOF) method for the dynamics of free boundaries. *Journal of computational physics*, 39(1), 201–225.

[15] Tong A. Y., & Wang Z. (2007). A numerical method for capillarity-dominant free surface flows. *Journal of Computational Physics*, 221(2), 506–523.

[16] Science Flow (2012). Version 12.0 user’s manual. Santa Fe, NM: Flow Science.

[17] Man C., Zhang G., Hong V., Zhou S., & Feng Y. (2019). Assessment of turbulence models on bridge-pier scour using Flow-3D. *World Journal of Engineering and Technology*, 7(2), 241–255.

[18] Yakhot V., & Smith L. M. (1992). The renormalization group, the ɛ-expansion and derivation of turbulence models. *Journal of scientific computing*, 7, 35–61.

[19] Patel V. C., Rodi W., & Scheuerer G. (1985). Turbulence models for near-wall and low Reynolds number flows-a review. *AIAA journal*, 23(9), 1308–1319.

[20] Soulsby R. L. (1997). Dynamics of marine sands: a manual for practical applications. Oceanographic Literature Review, 9(44), 947.

[21] Dey S. (2014). Bed-load transport. *Fluvial hydrodynamics*: *Hydrodynamic and sediment transport phenomena*, Berlin: Springer, 261–326.

[22] Cheng N. S., & Chiew Y. M. (1998). Pickup probability for sediment entrainment. *Journal of Hydraulic Engineering*, 124(2), 232–235.

[23] Melville B. W. (2000). *Bridge Scour* (Vol. 112). Water Resources Publications.

[24] Hosseini R., Fazloula R., Saneie M., & Amini A. (2018). Bagged neural network for estimating the scour depth around pile groups. *International Journal of River Basin Management*, 16(4), 401–412.

[25] Bohan J. P. (1970). *Erosion and Riprap Requirements at Culvert and Storm-drain Outlets*: *Hydraulic Laboratory Investigation*. Waterways Experiment Station.

[26] Amini A., & Parto A. A. (2017). 3D numerical simulation of flow field around twin piles. *Acta Geophysica*, 65(6), 1243–1251.

[27] Karami Moghadam M., Amini A., & Keshavarzi A. (2020). Intake design attributes and submerged vanes effects on sedimentation and shear stress. *Water and Environment Journal*, 34(3), 374–380.

[28] Day R. A., Liriano S. L., & White W. R. (2001, September). Effect of tailwater depth and model scale on scour at culvert outlets. In *Proceedings of the Institution of Civil Engineers-Water and Maritime Engineering* (Vol. 148, No. 3, pp. 189–198). Thomas Telford Ltd.

[29] Kim S. E., & Boysan F. (1999). Application of CFD to environmental flows. *Journal of Wind Engineering and Industrial Aerodynamics*, 81(1–3), 145–158.

[30] Hirsch C. (2007). *Numerical computation of internal and external flows*: *The fundamentals of computational fluid dynamics*. Elsevier.

[31] Biswas R., & Strawn R. C. (1998). Tetrahedral and hexahedral mesh adaptation for CFD problems. *Applied Numerical Mathematics*, 26(1–2), 135–151.

[32] Ecer, A., Fox, P., Periaux, J., Satofuka, N., & Keyes, D. E. (2000). Parallel Computational Fluid Dynamics: Towards Teraflops, Optimization, and Novel Formulations: Proceedings of the Parallel CFD’99 Conference.

[33] Eghbalzadeh A., Javan M., Hayati M., & Amini A. (2016). Discharge prediction of circular and rectangular side orifices using artificial neural networks. *KSCE Journal of Civil Engineering*, 20, 990–996.

