# Supplementary material for: Numerical modelling of downstream scour in circular culverts: Impact of inlet blockages and variable flow conditions
Source: PLoS One. 2024 Oct 31;19(10):e0312501. doi: 10.1371/journal.pone.0312501 (PMC11527307; doi:10.1371/journal.pone.0312501)
Supplement: S1 File — (PDF) [file pone.0312501.s001.pdf]

First Hydrograph; unsteady flow condition, 0% Blockage									
**Distance (mm)**	6 lit/s or 21.6 m3/h, **40 min**	10 lit/s or 36 m3/h, **80 min**	14 lit/s 50.4 m3/h, **120 min**	18 lit/s or 64.8 m3/h, **160 min**	22lit/s 79.2 m3/h, **200 min**	18 lit/s or 64.8 m3/h, **240 min**	14 lit/s 50.4 m3/h, **280 min**	10 lit/s or 36 m3/h, **320 min**	6 lit/s or 21.6 m3/h, **360 min**
**0**	-21.2	-22.8	-26.3	-32.3	-35.3	-39.0	-40.3	-44.6	-46.9
**20**	-35.6	-38.0	-39.9	-40.6	-45.3	-50.8	-52.7	-54.3	-57.0
**40**	-30.3	-38.0	-40.3	-41.0	-47.5	-51.4	-56.0	-58.5	-61.5
**60**	-41.0	-43.0	-44.6	-47.3	-52.6	-58.1	-60.4	-64.0	-66.7
**80**	-51.0	-54.4	-54.3	-58.6	-60.3	-65.0	-66.4	-62.3	-65.4
**100**	-56.5	-59.0	-61.0	-65.3	-69.7	-74.2	-76.6	-79.8	-79.8
**120**	-62.5	-65.4	-67.3	-70.2	-73.0	-78.1	-81.3	-83.2	-88.4
**140**	-61.6	-66.9	-68.7	-72.5	-75.3	-80.0	-85.3	-88.1	-91.0
**160**	-61.4	-67.9	-70.1	-76.3	-79.5	-83.2	-87.6	-90.9	-96.3
**180**	-61.5	-68.2	-73.5	-77.3	-84.9	-89.7	-90.5	-93.3	-98.1
**200**	-60.5	-69.0	-73.8	-82.6	-88.5	-92.1	-94.6	-97.8	-99.7
**220**	-59.6	-71.0	-74.7	-84.3	-89.6	-94.8	-97.9	-99.5	-100.5
**240**	-58.6	-73.0	-75.6	-86.3	-91.1	-94.3	-99.3	-102.6	-108.1
**260**	-56.8	-71.3	-76.0	-87.0	-92.6	-96.2	-99.7	-103.9	-106.8
**280**	-54.9	-71.0	-78.5	-89.0	-94.6	-98.5	-99.6	-105.9	-107.5
**300**	-56.2	-71.0	-77.7	-88.2	-95.1	-99.2	-100.3	-106.9	-108.8
**320**	-54.7	-68.3	-76.6	-87.6	-96.3	-100.7	-101.5	-105.3	-107.5
**340**	-51.0	-68.0	-74.3	-87.0	-95.6	-100.3	-102.6	-105.0	-106.8
**360**	-48.7	-68.4	-75.3	-85.2	-94.0	-101.0	-103.5	-104.4	-107.5
**380**	-47.0	-66.6	-74.0	-85.2	-94.0	-99.9	-102.2	-105.3	-106.6
**400**	-47.6	-64.0	-74.0	-84.0	-93.7	-98.3	-103.3	-104.3	-106.0
**420**	-47.6	-65.1	-73.2	-83.0	-90.7	-97.5	-102.2	-104.3	-105.3
**440**	-46.6	-66.4	-75.4	-83.0	-89.7	-99.3	-102.0	-103.5	-104.3
**460**	-42.6	-66.0	-72.7	-82.6	-88.4	-98.6	-100.3	-103.3	-105.3
**480**	-40.3	-62.5	-71.7	-83.3	-87.3	-96.3	-100.0	-101.3	-102.6
**500**	-39.7	-63.6	-68.7	-82.7	-83.6	-93.9	-98.9	-102.0	-103.4
**520**	-40.7	-63.0	-68.0	-82.0	-87.6	-90.7	-96.9	-101.0	-99.8
**540**	-38.7	-61.0	-67.5	-79.1	-86.6	-91.6	-95.3	-100.0	-100.6
**560**	-34.7	-59.3	-66.3	-77.2	-85.0	-90.9	-94.6	-98.0	-99.5
**580**	-33.0	-57.3	-65.1	-76.2	-81.5	-89.6	-92.6	-98.0	-99.6
**600**	-28.7	-53.7	-65.0	-75.2	-79.9	-87.2	-90.4	-95.9	-98.3
**620**	-25.0	-58.0	-63.8	-73.1	-78.6	-86.2	-89.7	-97.7	-99.4
**640**	-25.4	-50.7	-60.3	-72.0	-75.6	-80.6	-87.6	-94.0	-96.7
**660**	-22.8	-48.3	-59.6	-68.7	-75.0	-79.5	-86.3	-90.2	-94.0
**680**	-18.6	-46.6	-59.3	-65.3	-72.7	-76.6	-84.0	-93.1	-94.5
**700**	-17.3	-42.7	-58.8	-65.0	-69.9	-71.0	-81.1	-89.7	-90.7

**720**	-17.0	-39.0	-56.3	-63.1	-67.6	-69.6	-78.9	-86.3	-87.2
**740**	-14.9	-35.0	-55.9	-60.8	-63.6	-62.6	-76.3	-81.4	-88.3
**760**	-12.5	-30.6	-50.3	-58.6	-60.3	-62.6	-72.6	-83.5	-89.0
**780**	-10.2	-26.6	-48.3	-54.8	-59.7	-60.6	-69.0	-80.6	-86.8
**800**	-5.6	-20.7	-47.3	-51.5	-54.6	-59.9	-67.6	-75.3	-86.2
**820**	0.2	-16.0	-43.3	-49.0	-52.0	-60.3	-63.3	-72.6	-80.2
**840**	3.3	-12.6	-40.6	-44.0	-49.7	-58.7	-60.3	-70.3	-78.3
**860**	4.9	-10.7	-37.6	-44.0	-47.0	-56.6	-61.3	-68.0	-76.0
**880**	5.4	-9.7	-32.6	-41.0	-43.7	-52.7	-60.3	-66.3	-71.8
**900**	7.9	-7.6	-27.6	-39.5	-40.3	-50.3	-57.0	-68.8	-70.3
**920**	10.6	-5.4	-23.6	-36.2	-39.7	-45.9	-53.3	-61.3	-67.2
**940**	11.8	-2.5	-20.5	-32.5	-38.0	-40.6	-49.3	-56.3	-63.9
**960**	11.7	2.3	-18.6	-28.7	-30.6	-37.0	-43.3	-51.8	-59.0
**980**	10.4	9.7	-15.2	-25.3	-29.6	-32.6	-39.2	-49.6	-52.3
**1000**	8.6	12.6	-10.6	-21.0	-25.9	-29.6	-34.3	-45.1	-52.0
**1020**	7.4	15.9	-7.6	-17.9	-19.6	-22.4	-31.0	-46.4	-49.0
**1040**	5.7	16.6	-4.3	-14.6	-16.6	-19.0	-23.7	-37.6	-44.6
**1060**	5.3	17.5	2.6	-10.6	-12.6	-18.6	-24.2	-32.6	-40.8
**1080**	7.4	17.6	7.6	-7.7	-9.9	-17.5	-20.6	-28.9	-38.0
**1100**	2.1	17.0	8.6	-2.6	-7.5	-14.3	-18.4	-21.4	-38.2
**1120**	1.1	16.6	14.3	3.6	-2.6	-11.0	-15.3	-23.9	-35.0
**1140**		15.2	16.3	8.6	-1.2	-9.6	-12.3	-17.7	-32.7
**1160**		15.0	18.6	12.6	0.3	-7.6	-10.6	-15.2	-19.3
**1180**		13.6	18.9	15.6	8.7	-2.6	-9.3	-12.7	-15.3
**1200**		12.6	19.6	16.0	10.6	0.2	-7.9	-8.7	-10.2
**1220**		10.3	19.6	16.3	13.8	6.4	-2.6	-5.9	-8.3
**1240**		10.9	18.6	16.8	16.0	9.7	2.1	-3.9	-7.6
**1260**		9.7	18.3	17.9	18.8	12.6	8.6	-2.2	-5.3
**1280**		9.7	17.6	19.0	19.6	15.6	15.0	1.6	-2.7
**1300**		9.0	17.0	19.0	20.3	17.3	16.6	7.6	1.6
**1320**		8.5	16.0	19.1	19.9	19.0	19.6	10.3	8.5
**1340**		7.6	15.8	18.7	18.6	19.4	20.5	12.6	15.3
**1360**		7.0		17.3	17.6	20.6	21.6	16.3	17.6
**1380**		7.0		15.6	17.1	21.1	23.0	18.0	18.6
**1400**		6.5		14.3	16.5	20.3	22.6	18.8	19.6
**1420**		6.3		14.0	17.0	19.6	22.0	18.8	20.9
**1440**		5.6		13.1	14.6	19.0	21.5	20.1	22.0
**1460**		4.6		14.0	14.0	18.6	20.3	21.5	22.8
**1480**		4.6		14.3	13.6	17.6	19.3	22.5	22.2
**1500**		4.3			12.5	16.3	19.2	21.6	21.6
**1520**		4.2			11.6	15.2	18.7	20.6	20.3
**1540**		3.3						19.7	19.1
**1560**		5.6							18.7

**1580**		5.6							18.0
**1600**		4.6							17.0

First Hydrograph; unsteady flow condition, 15% Blockage									
**Distance (mm)**	6 lit/s or 21.6 m3/h, **40 min**	10 lit/s or 36 m3/h, **80 min**	14 lit/s 50.4 m3/h, **120 min**	18 lit/s or 64.8 m3/h, **160 min**	22lit/s 79.2 m3/h, **200 min**	18 lit/s or 64.8 m3/h, **240 min**	14 lit/s 50.4 m3/h, **280 min**	10 lit/s or 36 m3/h, **320 min**	6 lit/s or 21.6 m3/h, **360 min**
**0**	-27.3	-30.3	-32.6	-33.2	-36.8	-42.0	-44.1	-46.3	-47.7
**20**	-36.5	-45.3	-48.6	-52.0	-54.6	-60.6	-63.7	-66.8	-68.8
**40**	-48.3	-59.9	-60.5	-62.6	-70.7	-73.6	-78.3	-82.2	-84.6
**60**	-52.6	-72.8	-76.5	-81.4	-85.4	-86.6	-89.9	-94.4	-100.2
**80**	-58.8	-73.0	-79.3	-84.2	-90.4	-92.6	-99.2	-105.2	-110.3
**100**	-60.5	-78.5	-83.6	-86.7	-91.1	-95.6	-101.4	-105.5	-111.6
**120**	-63.6	-79.3	-85.4	-91.6	-96.2	-100.5	-106.6	-106.9	-110.1
**140**	-64.2	-84.6	-87.4	-92.7	-99.4	-98.6	-106.5	-108.8	-112.1
**160**	-63.3	-86.0	-90.6	-94.1	-100.8	-101.3	-105.3	-108.6	-114.8
**180**	-59.6	-87.2	-91.3	-97.8	-99.7	-103.6	-109.8	-110.3	-115.6
**200**	-58.6	-89.6	-92.7	-98.3	-101.2	-105.7	-110.9	-111.5	-116.8
**220**	-55.2	-88.6	-93.5	-99.2	-104.1	-107.9	-111.2	-111.8	-117.2
**240**	-52.7	-87.5	-94.6	-99.3	-104.3	-108.9	-112.4	-110.0	-116.3
**260**	-50.3	-83.7	-93.6	-98.2	-104.2	-110.7	-116.2	-114.0	-116.4
**280**	-45.3	-80.3	-92.7	-98.3	-104.2	-108.7	-114.1	-111.8	-115.1
**300**	-41.0	-78.3	-90.6	-97.1	-106.0	-107.9	-113.3	-112.9	-116.3
**320**	-39.2	-76.6	-92.6	-98.2	-106.1	-109.0	-114.4	-114.1	-114.5
**340**	-33.3	-75.3	-93.7	-99.3	-105.3	-109.6	-116.1	-117.9	-118.4
**360**	-32.8	-73.7	-92.0	-99.6	-104.6	-106.6	-115.9	-115.7	-119.2
**380**	-32.6	-70.6	-90.3	-98.8	-104.8	-108.8	-114.2	-114.9	-115.3
**400**	-31.6	-69.0	-84.2	-96.4	-102.2	-107.0	-113.3	-116.0	-117.5
**420**	-30.3	-65.3	-81.5	-94.6	-100.3	-104.6	-110.8	-114.4	-115.8
**440**	-29.6	-64.3	-81.5	-90.5	-98.0	-103.6	-111.8	-117.4	-117.9
**460**	-26.6	-66.4	-80.3	-84.3	-93.5	-101.2	-112.3	-115.9	-117.4
**480**	-21.0	-58.6	-79.6	-83.5	-91.7	-97.7	-110.5	-114.1	-114.5
**500**	-14.5	-56.0	-78.2	-86.2	-90.5	-99.0	-110.0	-112.5	-116.8
**520**	-14.0	-56.3	-74.7	-85.4	-90.7	-95.4	-108.1	-112.5	-116.9
**540**	-15.0	-53.7	-71.0	-82.5	-89.6	-96.4	-107.2	-110.5	-116.8
**560**	-10.4	-54.3	-68.3	-81.7	-88.8	-93.3	-103.9	-108.1	-116.4
**580**	-5.8	-53.7	-64.3	-78.5	-86.4	-91.0	-101.6	-106.7	-116.9
**600**	-4.2	-46.3	-60.3	-73.3	-83.9	-93.7	-104.3	-109.6	-116.8

**620**	-2.0	-40.3	-57.9	-70.8	-81.3	-90.7	-99.2	-110.1	-114.4
**640**	0.0	-35.6	-53.2	-65.9	-75.2	-89.7	-97.1	-111.0	-110.3
**660**	2.0	-37.6	-51.6	-62.2	-73.3	-86.3	-96.6	-110.4	-110.7
**680**	5.0	-36.6	-48.3	-60.7	-71.8	-83.2	-92.4	-107.0	-110.2
**700**	7.0	-32.6	-42.7	-50.8	-68.4	-79.5	-89.5	-104.0	-107.1
**720**	10.0	-29.6	-38.5	-50.4	-68.0	-78.7	-85.6	-102.9	-110.0
**740**	10.0	-25.3	-35.0	-46.7	-63.1	-75.9	-81.7	-99.8	-107.8
**760**	12.0	-22.0	-31.0	-38.5	-58.5	-72.0	-80.6	-96.6	-103.5
**780**	15.0	-22.6	-27.9	-32.2	-57.9	-67.3	-76.6	-94.4	-100.3
**800**	15.0	-20.3	-25.6	-30.9	-51.4	-62.7	-73.8	-91.5	-94.3
**820**	17.0	-19.0	-24.0	-30.2	-50.7	-58.6	-70.6	-87.1	-89.7
**840**	17.0	-19.0	-20.6	-31.6	-45.2	-54.2	-65.9	-79.2	-83.6
**860**	19.0	-16.3	-20.0	-28.0	-41.4	-50.0	-62.5	-75.6	-80.9
**880**	17.0	-15.8	-18.6	-26.5	-37.8	-48.5	-58.9	-71.9	-82.1
**900**	12.0	-14.3	-17.3	-26.1	-37.4	-49.5	-55.9	-68.7	-76.8
**920**	10.0	-10.3	-16.6	-24.4	-32.6	-46.3	-51.6	-64.2	-72.2
**940**	10.0	-8.6	-15.7	-20.4	-28.5	-41.6	-53.7	-66.4	-70.4
**960**	8.0	-3.5	-14.9	-15.6	-25.4	-38.4	-50.4	-62.9	-70.8
**980**	9.0	-2.5	-10.7	-11.2	-19.8	-32.6	-44.2	-56.4	-65.1
**1000**	8.0	0.2	-9.0	-10.4	-16.9	-27.9	-39.3	-41.3	-60.5
**1020**	9.0	1.3	-6.6	-9.9	-17.4	-24.3	-35.5	-37.3	-52.4
**1040**	10.0	6.6	-2.5	-6.7	-14.0	-20.9	-31.9	-39.5	-45.7
**1060**	8.0	10.6	2.7	-6.0	-13.3	-20.4	-30.4	-40.9	-42.1
**1080**	9.0	12.3	7.0	-3.5	-12.7	-16.3	-27.1	-38.4	-42.6
**1100**	7.0	12.3	9.7	-1.3	-10.3	-12.7	-23.3	-34.5	-35.5
**1120**	-5.0	11.3	12.5	0.3	-9.3	-9.6	-20.1	-31.1	-32.1
**1140**	3.0	10.3	13.6	6.9	-5.2	-6.0	-18.3	-27.2	-31.0
**1160**	2.0	9.3	14.3	10.3	-3.8	-2.7	-14.8	-24.5	-32.3
**1180**	1.0	7.6	14.3	11.9	0.5	0.3	-9.3	-19.7	-30.3
**1200**		6.6	14.0	13.6	3.3	7.0	-7.3	-15.7	-16.2
**1220**		6.0	12.6	15.0	8.7	13.5	-3.3	-10.4	-10.7
**1240**			13.0	14.7	12.4	16.6	-1.3	-9.3	-9.6
**1260**					15.3	18.0	3.7	3.8	3.9
**1280**						19.7	9.0	9.4	9.7
**1300**						21.0	12.6	13.2	13.6
**1320**						21.6	17.6	18.5	19.0
**1340**						21.6	19.5	20.5	21.1

First Hydrograph; unsteady flow condition, 30% Blockage									
**Distance (mm)**	6 lit/s or 21.6 m3/h, **40 min**	10 lit/s or 36 m3/h, **80 min**	14 lit/s 50.4 m3/h, **120 min**	18 lit/s or 64.8 m3/h, **160 min**	22lit/s 79.2 m3/h, **200 min**	18 lit/s or 64.8 m3/h, **240 min**	14 lit/s 50.4 m3/h, **280 min**	10 lit/s or 36 m3/h, **320 min**	6 lit/s or 21.6 m3/h, **360 min**
**0**	-42.3	-45.3	-50.4	-55.6	-57.5	-60.6	-60.3	-61.5	-61.6
**20**	-52.5	-55.4	-57.3	-59.3	-61.3	-65.9	-66.6	-67.9	-68.3
**40**	-58.5	-62.7	-65.4	-69.4	-70.3	-73.6	-72.7	-74.0	-75.7
**60**	-54.9	-66.3	-70.2	-75.3	-81.4	-81.3	-80.3	-82.7	-83.5
**80**	-64.4	-70.2	-77.4	-77.2	-87.6	-90.6	-85.6	-88.0	-86.0
**100**	-65.5	-73.7	-84.5	-91.0	-94.3	-92.7	-90.5	-92.7	-92.6
**120**	-66.3	-76.3	-92.3	-96.2	-97.3	-98.6	-99.4	-100.3	-101.6
**140**	-70.3	-77.3	-98.4	-97.4	-96.3	-102.3	-105.6	-106.2	-107.6
**160**	-73.3	-80.2	-99.1	-98.3	-98.5	-102.6	-103.5	-107.3	-107.6
**180**	-69.0	-83.0	-99.6	-100.2	-100.3	-107.6	-105.2	-108.7	-109.6
**200**	-66.6	-83.0	-98.3	-101.2	-105.3	-109.6	-105.6	-109.8	-110.3
**220**	-50.6	-86.3	-96.0	-100.4	-107.6	-110.7	-112.3	-114.3	-115.4
**240**	-45.3	-86.0	-97.3	-99.6	-109.7	-112.3	-111.3	-116.0	-117.6
**260**	-39.0	-81.3	-96.0	-98.3	-110.2	-115.2	-116.5	-116.3	-117.0
**280**	-38.2	-74.0	-91.2	-98.5	-109.5	-115.5	-117.3	-119.0	-119.4
**300**	-36.3	-70.0	-87.2	-96.3	-107.6	-117.0	-118.3	-119.2	-120.4
**320**	-38.0	-64.7	-83.7	-91.3	-103.0	-114.9	-119.4	-124.7	-125.6
**340**	-33.8	-63.0	-80.7	-87.0	-97.3	-113.7	-121.0	-125.7	-126.0
**360**	-39.6	-62.5	-78.4	-82.7	-91.6	-111.0	-119.6	-125.5	-126.8
**380**	-38.0	-60.0	-76.3	-82.7	-87.3	-107.6	-118.8	-124.6	-124.5
**400**	-31.6	-52.1	-76.0	-80.4	-84.0	-100.3	-115.2	-124.5	-125.4
**420**	-27.0	-53.0	-75.0	-84.0	-80.4	-102.5	-112.4	-122.7	-125.6
**440**	-20.4	-55.0	-62.6	-81.0	-77.2	-100.7	-113.3	-118.7	-126.0
**460**	-17.6	-48.0	-68.4	-79.7	-74.0	-96.5	-112.3	-116.0	-125.2
**480**	-8.5	-45.2	-63.6	-77.7	-70.7	-90.3	-108.6	-116.6	-123.3
**500**	-8.5	-42.7	-59.1	-69.7	-69.0	-85.5	-101.6	-112.1	-120.5
**520**	-5.1	-36.8	-53.6	-66.4	-66.9	-79.6	-95.3	-106.6	-115.2
**540**	-3.4	-35.7	-54.3	-63.9	-66.3	-81.5	-89.3	-99.6	-110.2
**560**	-1.7	-38.0	-53.3	-59.9	-62.9	-81.0	-82.7	-90.3	-108.6
**580**	0.0	-32.0	-50.4	-54.9	-59.7	-72.6	-78.3	-88.5	-101.0
**600**	0.0	-26.0	-47.3	-50.4	-56.9	-68.5	-76.3	-82.7	-96.4
**620**	1.2	-27.7	-40.3	-49.0	-52.8	-62.6	-70.1	-80.6	-90.3
**640**	3.7	-24.0	-42.4	-45.3	-51.0	-60.4	-69.0	-79.7	-88.7
**660**	5.3	-21.5	-32.6	-40.9	-46.3	-60.4	-67.0	-78.4	-80.6
**680**	5.7	-14.6	-23.0	-38.0	-40.6	-58.5	-60.9	-77.0	-80.4
**700**	5.6	-10.8	-23.6	-30.7	-36.6	-50.7	-58.4	-70.2	-78.4

**720**	6.0	-6.0	-20.8	-27.6	-30.6	-46.3	-53.8	-65.3	-70.7
**740**	6.0	-3.0	-16.3	-21.0	-27.6	-40.6	-50.7	-61.0	-64.1
**760**	6.0	0.0	-12.8	-15.3	-20.5	-39.0	-48.0	-55.5	-61.0
**780**	6.5	0.0	-8.0	-10.7	-15.2	-36.5	-40.3	-50.1	-62.5
**800**	5.7	0.2	-2.4	-6.0	-10.2	-30.6	-32.5	-43.9	-60.0
**820**	5.6	2.7	0.6	-0.7	-6.3	-28.5	-29.7	-41.5	-56.2
**840**	6.4	4.0	3.3	1.6	-2.5	-28.6	-28.6	-38.6	-50.1
**860**	5.2	4.0	4.3	4.3	-1.2	-23.6	-25.7	-32.6	-46.2
**880**	5.7	3.9	6.0	7.9	1.0	-18.6	-22.7	-30.6	-40.8
**900**	4.3	4.5	6.2	9.0	4.9	-10.6	-19.9	-28.5	-35.2
**920**	4.0	5.0	7.0	9.5	8.5	-8.7	-16.6	-24.0	-30.3
**940**	6.5	5.1	9.7	10.9	10.5	-5.2	-10.3	-19.5	-24.2
**960**	5.6	5.3	10.3	11.8	12.6	-0.3	-7.3	-18.6	-20.6
**980**	2.6	5.7	11.0	12.6	14.8	4.3	-2.7	-16.2	-19.5
**1000**	5.7	6.3	11.0	14.0	14.6	5.2	0.6	-11.2	-17.3
**1020**	6.5	6.3	11.2	13.3	16.0	6.6	6.0	-9.0	-12.6
**1040**	5.2	7.3	8.3	14.3	15.3	10.3	7.2	-4.2	-10.8
**1060**	3.9	8.0	9.7	15.0	17.0	12.6	10.4	-0.3	-9.2
**1080**	2.6	7.9	11.3	15.5	17.5	16.5	14.3	2.2	-2.5
**1100**		8.4	12.7	15.3	18.0	17.9	17.0	8.3	2.3
**1120**		8.1	13.3	16.6	16.2	19.3	17.3	11.5	7.6
**1140**		9.6	14.0	17.0	17.3	18.6	19.4	15.2	11.0
**1160**		10.0	14.6	15.8	16.3	17.6	19.6	16.6	12.5
**1180**		10.2	15.0	15.0	15.2	17.0	20.6	18.2	12.5
**1200**		9.6	13.3	15.0	16.3	16.6	21.5	20.3	16.0
**1220**		9.0	12.7	16.6	15.2	17.0	21.0	21.6	16.5
**1240**		8.6	12.0	14.3	14.3	15.7	19.6	22.7	17.5
**1260**		8.8	11.5	14.2	15.0	14.9	20.4	22.0	19.0
**1280**		7.3	11.0	13.6	15.0	15.9	20.0	21.6	19.3
**1300**		6.5	11.0	13.0	16.0	14.0	19.9	20.7	20.8
**1320**		4.3		13.0	15.0	14.0	19.8	20.1	21.0
**1340**		5.0		12.3	13.0	12.2	19.1	19.3	21.3
**1360**		5.2		11.4		12.5	18.6	19.0	21.9
**1380**		5.0		11.0		14.0	17.5	19.0	22.6
**1400**		4.6				13.5	17.4	17.0	23.0
**1420**		3.3					17.0	16.3	20.2
**1440**		3.9					16.2	15.6	20.0
**1460**		3.3					17.5	17.0	19.4
**1480**							16.3	15.9	19.8
**1500**							16.0		18.5
**1520**							15.8		18.0
**1540**									17.3

Second Hydrograph; unsteady flow condition, 0% Blockage									
**Distance (mm)**	2 lit/s or 7.2 m3/h, **25 min**	5 lit/s or 18 m3/h, **50 min**	8 lit/s 28.8 m3/h, **75 min**	11 lit/s or 39.6 m3/h, **100 min**	14 lit/s 50.4 m3/h, **125 min**	11 lit/s or 39.6 m3/h, **150 min**	8 lit/s 28.8 m3/h, **175 min**	5 lit/s or 18 m3/h, **200 min**	2 lit/s or 7.2 m3/h, **225 min**
**0**	-17.0	-20.9	-25.6	-22.9	-30.1	-26.4	-28.1	-29.1	-30.1
**20**	-24.4	-44.4	-49.8	-49.8	-45.2	-50.2	-62.2	-63.3	-65.3
**40**	-37.3	-67.5	-73.4	-73.0	-48.2	-64.5	-52.2	-53.2	-54.2
**60**	-37.3	-69.5	-78.7	-77.9	-55.2	-55.2	-55.2	-57.2	-57.9
**80**	-41.1	-70.2	-80.6	-84.2	-63.5	-61.5	-60.8	-61.2	-61.4
**100**	-42.8	-72.0	-81.3	-82.6	-53.2	-72.5	-70.7	-71.3	-71.8
**120**	-40.5	-73.5	-82.3	-83.5	-62.6	-78.5	-76.7	-77.8	-78.8
**140**	-37.1	-75.5	-82.0	-84.7	-73.2	-82.8	-81.6	-82.2	-82.8
**160**	-29.8	-78.2	-82.0	-83.2	-79.3	-86.8	-88.0	-89.4	-90.4
**180**	-28.9	-79.5	-81.1	-83.0	-85.8	-89.6	-88.4	-90.4	-92.4
**200**	-23.5	-76.0	-79.4	-82.7	-85.4	-88.8	-95.4	-95.9	-96.4
**220**	-23.1	-71.9	-78.3	-83.5	-85.0	-88.2	-96.0	-96.3	-98.4
**240**	-17.9	-68.9	-73.1	-83.7	-84.5	-86.3	-95.0	-97.0	-98.3
**260**	-12.6	-61.9	-68.5	-82.5	-85.9	-85.2	-95.4	-96.0	-97.3
**280**	-5.2	-57.6	-63.0	-77.3	-89.8	-81.8	-92.4	-95.4	-97.4
**300**	-5.2	-50.4	-61.1	-77.3	-84.5	-76.3	-82.0	-82.5	-85.3
**320**	-13.6	-38.7	-54.9	-73.0	-79.5	-75.8	-79.3	-80.2	-82.3
**340**	-13.6	-32.9	-56.1	-66.0	-79.5	-72.4	-73.7	-74.8	-76.8
**360**	-10.4	-28.2	-46.1	-59.9	-78.0	-69.3	-76.5	-77.3	-78.8
**380**	-7.3	-16.9	-46.0	-57.4	-72.7	-65.3	-70.5	-71.5	-79.3
**400**	-6.3	-10.4	-38.3	-55.4	-68.3	-60.2	-72.6	-73.3	-78.0
**420**	-5.2	-5.2	-39.5	-46.5	-62.0	-66.5	-66.6	-67.3	-70.3
**440**	-3.1	0.0	-39.6	-44.0	-69.2	-63.0	-60.6	-61.2	-62.8
**460**	0.0	0.0	-33.2	-43.0	-63.1	-59.2	-67.7	-69.1	-70.3
**480**	0.0	1.0	-26.9	-43.2	-62.0	-55.5	-61.6	-62.4	-63.2
**500**	2.1	3.1	-23.3	-37.7	-57.6	-49.0	-61.3	-62.4	-63.8
**520**	4.2	5.2	-21.4	-37.9	-55.9	-50.2	-55.6	-59.2	-63.3
**540**	5.2	6.3	-20.9	-26.1	-55.4	-49.2	-57.3	-57.2	-58.7
**560**	5.2	7.3	-20.4	-21.3	-52.9	-44.5	-48.6	-50.2	-52.2
**580**	7.3	10.4	-15.1	-20.1	-51.6	-42.9	-48.9	-49.2	-50.1
**600**	9.4	9.4	-9.6	-16.1	-46.7	-38.1	-49.9	-52.2	-53.4
**620**	9.4	5.2	-10.4	-15.6	-44.7	-26.8	-46.2	-48.2	-49.1
**640**	10.4	8.4	-10.9	-5.0	-39.9	-29.1	-42.9	-43.4	-45.7
**660**	9.4	7.3	-10.2	-3.0	-36.3	-26.4	-37.0	-37.7	-38.7

**680**	8.4	7.3	-5.2	-1.0	-29.3	-30.1	-32.4	-34.6	-35.6
**700**	6.3	6.3	-3.1		-29.7	-28.5	-27.1	-28.1	-30.0
**720**	2.1	3.1	-1.0		-28.4	-24.3	-27.1	-27.6	-28.3
**740**	5.2	2.1			-27.4	-16.5	-27.6	-28.1	-28.6
**760**	1.0	1.0			-29.4	-16.1	-24.7	-25.1	-25.7
**780**	1.0				-26.4	-16.3	-26.5	-27.1	-28.9
**800**					-19.8	-10.4	-21.5	-21.9	-21.6
**820**					-14.3	-11.5	-16.0	-16.1	-18.1
**840**					-13.3	-11.5	-14.4	-15.6	-17.1
**860**					-12.6	-10.0	-12.0	-13.4	-15.1
**880**					-11.5	-12.0	-11.0	-11.5	-14.1
**900**					-18.0	-9.0	-10.0	-14.1	-19.1
**920**					-12.3	-8.0	-7.0	-12.0	-18.1
**940**					-11.9	-5.0	-5.0	-11.0	-15.1
**960**					-11.5	-3.0	-1.0	-10.0	-12.0
**980**					-11.0	0.0	0.0	-9.0	-10.0
**1000**					-10.0	2.0	0.0	-10.0	-8.0
**1020**					-8.0	5.0	5.0	-8.0	-8.0
**1040**					-9.0	8.0	4.0	-7.0	-9.0
**1060**					-7.0	9.0	9.0	-5.0	-1.0
**1080**					-3.0	10.0	10.0	-2.0	0.0
**1100**					-2.0	9.0	8.0	0.0	0.0
**1120**					-1.0	8.0	7.0	3.0	2.0
**1140**						7.0	5.0	5.0	5.0
**1160**						5.0	5.0	8.0	9.0
**1180**						3.0	3.0	9.0	8.0
**1200**						2.0	2.0	11.0	14.1
**1220**						1.0		13.1	12.0
**1240**								12.0	15.1
**1260**								11.0	18.1
**1280**								10.0	17.1
**1300**								9.0	15.1
**1320**								8.0	12.0
**1340**								7.0	12.0
**1360**								5.0	14.1
**1380**								2.0	10.0
**1400**									9.0
**1420**									8.0
**1440**									4.0
**1460**									5.0
**1480**									2.0

Second Hydrograph; unsteady flow condition, 15% Blockage									
**Distance (mm)**	2 lit/s or 7.2 m3/h, **25 min**	5 lit/s or 18 m3/h, **50 min**	8 lit/s 28.8 m3/h, **75 min**	11 lit/s or 39.6 m3/h, **100 min**	14 lit/s 50.4 m3/h, **125 min**	11 lit/s or 39.6 m3/h, **150 min**	8 lit/s 28.8 m3/h, **175 min**	5 lit/s or 18 m3/h, **200 min**	2 lit/s or 7.2 m3/h, **225 min**
**0**	-5.2	-9.4	-12.5	-15.6	-22.9	-29.2	-31.3	-36.5	-41.7
**20**	-14.6	-25.0	-36.5	-46.9	-52.2	-60.5	-62.6	-64.7	-67.8
**40**	-20.9	-38.6	-64.7	-73.0	-75.1	-78.2	-79.3	-78.2	-81.4
**60**	-29.2	-46.9	-75.1	-83.4	-85.5	-88.7	-89.7	-93.9	-97.0
**80**	-35.5	-54.2	-81.4	-88.7	-90.7	-93.9	-96.0	-97.0	-100.1
**100**	-35.5	-64.7	-83.4	-93.9	-97.0	-96.0	-98.0	-100.1	-103.3
**120**	-36.5	-70.9	-88.7	-96.0	-98.0	-99.1	-101.2	-103.3	-104.3
**140**	-38.6	-75.1	-91.8	-97.0	-97.0	-100.1	-102.2	-104.3	-106.4
**160**	-37.5	-77.2	-91.8	-99.1	-98.0	-101.2	-103.3	-106.4	-107.4
**180**	-36.5	-78.2	-92.8	-98.0	-99.1	-103.5	-105.3	-108.5	-110.6
**200**	-34.4	-75.1	-89.7	-97.0	-101.2	-103.3	-107.4	-108.5	-110.6
**220**	-33.4	-70.9	-91.8	-99.2	-102.2	-104.3	-108.5	-110.6	-111.6
**240**	-33.4	-65.7	-88.7	-97.0	-103.3	-105.1	-109.5	-110.6	-111.6
**260**	-31.3	-63.6	-85.5	-98.0	-101.2	-105.3	-107.4	-108.5	-110.6
**280**	-30.2	-61.5	-83.4	-96.0	-100.1	-106.4	-108.5	-109.5	-111.6
**300**	-28.2	-59.5	-81.4	-93.9	-99.1	-104.3	-109.6	-111.6	-112.6
**320**	-21.9	-58.4	-82.4	-90.7	-97.0	-103.3	-108.5	-110.6	-113.7
**340**	-19.8	-57.4	-81.4	-89.7	-97.0	-104.3	-106.4	-110.6	-110.6
**360**	-18.8	-52.2	-79.3	-87.6	-98.0	-104.3	-106.4	-110.6	-111.6
**380**	-15.6	-50.1	-78.2	-86.6	-96.0	-103.3	-104.3	-108.5	-110.6
**400**	-12.5	-43.8	-73.0	-86.6	-96.0	-102.2	-104.3	-107.4	-110.6
**420**	-7.3	-39.6	-72.0	-85.5	-93.9	-101.2	-103.3	-107.4	-109.5
**440**	-6.3	-36.5	-70.9	-84.5	-92.8	-100.1	-103.3	-106.4	-109.5
**460**	-5.2	-31.3	-68.8	-88.3	-90.7	-99.1	-103.3	-106.4	-108.5
**480**	-2.1	-28.2	-67.8	-85.0	-91.8	-98.0	-102.2	-105.3	-107.4
**500**	0.0	-25.0	-62.6	-83.9	-89.7	-97.0	-100.1	-103.3	-105.3
**520**	1.0	-18.8	-69.5	-82.7	-88.7	-97.0	-99.1	-104.3	-106.4
**540**	2.1	-16.7	-67.1	-79.4	-87.6	-98.0	-101.2	-104.3	-107.4
**560**	2.1	-14.6	-64.7	-76.0	-83.4	-97.0	-101.2	-103.3	-106.4
**580**	2.1	-14.6	-61.1	-69.3	-79.3	-93.9	-99.1	-102.2	-104.3
**600**	3.1	-9.4	-56.3	-66.0	-77.2	-90.7	-97.0	-101.2	-103.3
**620**	0.0	-7.3	-50.3	-60.4	-76.1	-87.6	-98.0	-101.2	-104.3
**640**		-2.1	-44.3	-60.4	-72.0	-84.5	-98.0	-102.2	-105.3
**660**		0.0	-38.4	-55.9	-66.8	-81.4	-96.0	-97.0	-102.2
**680**		1.0	-33.6	-53.7	-62.6	-82.4	-90.7	-96.0	-101.2

**700**		2.1	-28.8	-50.3	-62.6	-79.3	-91.8	-94.9	-99.1
**720**		2.1	-21.6	-45.8	-60.5	-75.1	-87.6	-93.9	-97.0
**740**		3.1	-18.0	-42.5	-56.3	-70.9	-83.4	-90.7	-96.0
**760**		5.2	-12.0	-35.8	-52.2	-62.6	-79.3	-87.6	-93.9
**780**		6.3	-4.8	-31.3	-49.0	-59.5	-73.0	-82.4	-89.7
**800**		6.3	0.0	-24.6	-44.8	-57.4	-69.9	-76.1	-83.4
**820**		5.2	1.2	-17.9	-43.8	-52.2	-62.6	-70.9	-79.3
**840**		6.3	2.2	-11.2	-39.6	-49.0	-56.3	-64.7	-73.0
**860**		4.2	2.1	-8.9	-31.3	-43.8	-52.2	-62.6	-70.9
**880**		3.1	3.1	-2.1	-27.1	-38.6	-46.9	-60.5	-69.9
**900**		0.0	6.3	0.0	-20.9	-33.4	-41.7	-54.2	-66.8
**920**			6.3	1.0	-14.6	-29.2	-41.7	-49.0	-60.5
**940**			7.3	3.1	-8.3	-25.0	-39.6	-43.8	-52.2
**960**			8.3	5.2	-4.2	-21.9	-36.5	-41.7	-54.2
**980**			8.3	8.3	-2.1	-20.9	-31.3	-38.6	-44.8
**1000**			6.3	9.4	0.0	-17.7	-26.1	-33.4	-40.7
**1020**			8.3	11.5	4.2	-14.6	-20.9	-29.2	-36.5
**1040**			6.3	11.5	7.3	-12.5	-18.8	-28.2	-33.4
**1060**			7.3	11.5	8.3	-10.4	-15.6	-27.1	-33.4
**1080**			6.3	11.5	11.5	-5.2	-12.5	-22.9	-31.3
**1100**			5.2	11.5	13.6	0.0	-5.2	-17.7	-28.2
**1120**			2.1	9.4	15.6	1.0	0.0	-12.5	-22.9
**1140**			0.0	10.4	15.6	5.2	1.0	-9.4	-19.8
**1160**				8.3	15.6	7.3	5.2	-4.2	-15.6
**1180**				7.3	15.6	8.3	7.3	-2.1	-12.5
**1200**				6.3	15.6	12.5	9.4	0.0	-9.4
**1220**				5.2	14.6	15.6	12.5	5.2	-8.3
**1240**				0.0	12.5	16.7	15.6	9.4	-5.2
**1260**					11.5	17.7	16.7	15.6	0.0
**1280**					9.4	17.7	18.8	15.6	7.3
**1300**					8.3	16.7	18.8	18.8	12.5
**1320**					5.2	15.6	18.8	18.8	14.6
**1340**					2.1	14.6	16.7	17.7	16.7
**1360**					0.0	13.6	15.6	16.7	19.8
**1380**						12.5	14.6		20.9
**1400**						10.4	13.6		20.9
**1420**						9.4	14.6		19.8
**1440**						8.3	12.5		18.8
**1460**						5.2	10.4		18.8
**1480**						3.1	11.5		17.7
**1500**						0			16.688

Second Hydrograph; unsteady flow condition, 30% Blockage									
**Distance (mm)**	2 lit/s or 7.2 m3/h, **25 min**	5 lit/s or 18 m3/h, **50 min**	8 lit/s 28.8 m3/h, **75 min**	11 lit/s or 39.6 m3/h, **100 min**	14 lit/s 50.4 m3/h, **125 min**	11 lit/s or 39.6 m3/h, **150 min**	8 lit/s 28.8 m3/h, **175 min**	5 lit/s or 18 m3/h, **200 min**	2 lit/s or 7.2 m3/h, **225 min**
**0**	-4.2	-10.4	-20.8	-33.3	-41.6	-44.8	-52.1	-59.3	-62.5
**20**	-14.6	-31.2	-41.6	-43.7	-54.1	-65.6	-71.8	-65.6	-67.7
**40**	-22.9	-43.7	-54.1	-62.5	-64.5	-67.7	-72.9	-76.0	-72.9
**60**	-30.2	-48.9	-60.4	-66.6	-69.7	-72.9	-77.0	-80.2	-81.2
**80**	-36.4	-59.3	-66.6	-72.9	-75.0	-78.1	-82.2	-86.4	-88.5
**100**	-36.4	-61.4	-70.8	-81.2	-83.3	-85.4	-89.5	-88.5	-92.6
**120**	-37.5	-64.5	-71.8	-86.4	-89.5	-92.6	-95.8	-93.7	-96.8
**140**	-36.4	-70.8	-78.1	-88.5	-95.8	-98.9	-99.9	-96.8	-98.9
**160**	-34.4	-67.7	-85.4	-92.6	-101.0	-103.1	-106.2	-101.0	-103.1
**180**	-35.4	-69.7	-90.6	-96.8	-103.1	-107.2	-109.3	-104.1	-106.2
**200**	-34.4	-75.0	-95.8	-98.9	-104.1	-109.3	-111.4	-109.3	-108.3
**220**	-33.3	-81.2	-102.0	-104.1	-109.3	-105.1	-110.3	-111.4	-108.3
**240**	-31.2	-82.2	-106.2	-105.1	-110.3	-104.1	-113.5	-111.4	-109.3
**260**	-31.2	-85.4	-109.3	-107.2	-108.3	-109.3	-108.3	-115.6	-112.4
**280**	-29.1	-84.3	-104.1	-111.4	-113.5	-111.4	-113.5	-117.6	-115.6
**300**	-28.1	-83.3	-98.9	-107.2	-116.6	-119.7	-114.5	-120.8	-116.6
**320**	-28.1	-82.2	-83.3	-102.0	-115.6	-116.6	-116.6	-122.8	-119.7
**340**	-27.1	-81.2	-79.1	-97.9	-112.4	-114.5	-121.8	-122.8	-120.8
**360**	-26.0	-62.5	-75.0	-93.7	-110.3	-109.3	-119.7	-123.9	-122.8
**380**	-23.9	-60.4	-70.8	-90.6	-104.1	-104.1	-120.8	-121.8	-126.0
**400**	-19.8	-54.1	-67.7	-86.4	-95.8	-102.0	-113.5	-118.7	-122.8
**420**	-15.6	-52.1	-62.5	-79.1	-90.6	-98.9	-106.2	-114.5	-118.7
**440**	-14.6	-50.0	-60.4	-69.7	-88.5	-93.7	-105.1	-108.3	-114.5
**460**	-13.5	-47.9	-58.3	-67.7	-79.1	-90.6	-103.1	-106.2	-110.3
**480**	-12.5	-43.7	-55.2	-62.5	-72.9	-83.3	-97.9	-103.1	-107.2
**500**	-11.5	-39.6	-52.1	-63.5	-69.7	-78.1	-93.7	-99.9	-106.2
**520**	-8.3	-35.4	-50.0	-59.3	-66.6	-78.1	-89.5	-95.8	-104.1
**540**	-5.2	-29.1	-47.9	-53.1	-62.5	-72.9	-83.3	-93.7	-101.0
**560**	0.0	-25.0	-43.7	-48.9	-59.3	-67.7	-84.3	-91.6	-99.9
**580**	1.0	-22.9	-40.6	-46.8	-55.2	-65.6	-79.1	-89.5	-95.8
**600**	2.1	-18.7	-33.3	-41.6	-52.1	-61.4	-75.0	-83.3	-91.6
**620**	2.1	-15.6	-32.3	-39.6	-53.1	-61.4	-69.7	-81.2	-90.6
**640**	2.1	-14.6	-29.1	-35.4	-51.0	-59.3	-66.6	-77.0	-88.5
**660**	3.1	-10.4	-26.0	-33.3	-46.8	-56.2	-65.6	-72.9	-83.3

**680**	3.1	-8.3	-21.9	-31.2	-41.6	-52.1	-66.6	-70.8	-79.1
**700**	4.2	-4.2	-19.8	-29.1	-38.5	-50.0	-62.5	-65.6	-76.0
**720**	4.2	0.0	-15.6	-22.9	-31.2	-48.9	-64.5	-67.7	-72.9
**740**	5.2	4.2	-12.5	-17.7	-28.1	-44.8	-60.4	-64.5	-70.8
**760**	4.2	7.3	-8.3	-14.6	-25.0	-39.6	-56.2	-59.3	-66.6
**780**	4.2	8.3	0.0	-12.5	-19.8	-33.3	-50.0	-54.1	-62.5
**800**	3.1	9.4	6.2	-10.4	-19.8	-30.2	-41.6	-50.0	-59.3
**820**	3.1	9.4	8.3	-9.4	-15.6	-25.0	-33.3	-47.9	-52.1
**840**	0.0	9.4	9.4	-5.2	-12.5	-20.8	-31.2	-41.6	-48.9
**860**		10.4	12.5	-1.0	-8.3	-18.7	-28.1	-39.6	-46.8
**880**		8.3	12.5	0.0	-9.4	-14.6	-20.8	-37.5	-43.7
**900**		7.3	12.5	5.2	-5.2	-12.5	-17.7	-35.4	-41.6
**920**		6.2	12.5	8.3	0.0	-10.4	-14.6	-31.2	-39.6
**940**		5.2	10.4	12.5	4.2	-5.2	-8.3	-27.1	-37.5
**960**		4.2	10.4	15.6	7.3	0.0	-5.2	-25.0	-36.4
**980**			9.4	14.6	9.4	5.2	-3.1	-20.8	-31.2
**1000**			9.4	16.7	14.6	10.4	0.0	-18.7	-29.1
**1020**			8.3	16.7	16.7	16.7	5.2	-15.6	-25.0
**1040**			8.3	16.7	16.7	18.7	9.4	-12.5	-27.1
**1060**			7.3	15.6	15.6	18.7	12.5	-8.3	-20.8
**1080**			5.2	14.6	16.7	18.7	15.6	-5.2	-18.7
**1100**				14.6	15.6	16.7	18.7	-2.1	-16.7
**1120**				13.5	14.6	17.7	18.7	0.0	-14.6
**1140**				12.5	13.5	17.7	18.9	2.1	-12.5
**1160**				11.5	12.5	16.7	19.8	5.2	-8.3
**1180**				10.4	11.5	15.6	18.7	9.4	-9.4
**1200**				9.4	10.4	14.6	17.7	18.7	-5.2
**1220**				8.3	10.4	13.5	17.7	17.7	0.0
**1240**				5.2	8.3	12.5	16.7	18.7	5.2
**1260**					9.4	10.4	15.6	15.6	7.3
**1280**					7.3	8.3	12.5	16.7	9.4
**1300**					6.2	8.3	10.4	14.6	12.5
**1320**					6.2	8.3	9.4	12.5	15.6
**1340**					6.2	8.3	9.4	10.4	18.7
**1360**					6.2		9.4	10.4	18.7
**1380**							9.4	10.4	19.8
**1400**							9.4	9.4	19.8
**1420**								9.4	18.7
**1440**									16.7
**1460**									15.6
**1480**									12.5

First Hydrograph, Steady flow condition; 360 min, Q_m=22 l/s					
0% Blockage		15% Blockage		30% Blockage	
Distance (mm)	Scour depth (mm)	Distance (mm)	Scour depth (mm)	Distance (mm)	Scour depth (mm)
**0**	-5.15	0	-63.86	0	-34.505
**20**	-72.1	20	-87.55	20	-46.144
**40**	-77.25	40	-82.4	40	-50.573
**60**	-72.1	60	-74.16	60	-58.607
**80**	-70.04	80	-74.16	80	-61.388
**100**	-70.04	100	-75.19	100	-62.83
**120**	-74.16	120	-76.22	120	-64.89
**140**	-77.25	140	-76.941	140	-74.16
**160**	-82.4	160	-81.267	160	-80.546
**180**	-84.46	180	-85.49	180	-84.975
**200**	-87.55	200	-87.55	200	-91.258
**220**	-91.67	220	-92.7	220	-93.936
**240**	-94.76	240	-93.73	240	-98.365
**260**	-99.91	260	-95.79	260	-97.026
**280**	-97.85	280	-92.906	280	-98.056
**300**	-95.79	300	-96.82	300	-99.189
**320**	-99.91	320	-99.91	320	-101.97
**340**	-105.06	340	-105.06	340	-97.85
**360**	-104.03	360	-108.15	360	-97.129
**380**	-103	380	-109.18	380	-92.7
**400**	-100.94	400	-114.33	400	-90.228
**420**	-104.03	420	-112.27	420	-91.67
**440**	-103	440	-114.33	440	-82.091
**460**	-107.12	460	-115.36	460	-79.413
**480**	-108.15	480	-121.54	480	-80.34
**500**	-110.21	500	-123.6	500	-73.439
**520**	-113.5	520	-122.57	520	-66.95
**540**	-113.3	540	-124.63	540	-67.98
**560**	-111.24	560	-120.51	560	-64.89
**580**	-104.03	580	-118.45	580	-61.8
**600**	-103	600	-118.45	600	-60.77

**620**	-101.97	620	-117.42	620	-62.83
**640**	-94.76	640	-114.33	640	-63.86
**660**	-99.91	660	-112.27	660	-61.8
**680**	-97.85	680	-115.36	680	-61.8
**700**	-92.7	700	-114.33	700	-60.77
**720**	-90.64	720	-111.24	720	-58.71
**740**	-86.52	740	-112.27	740	-53.56
**760**	-80.34	760	-106.09	760	-58.71
**780**	-82.4	780	-100.94	780	-55.62
**800**	-78.28	800	-97.85	800	-51.5
**820**	-72.1	820	-93.73	820	-49.44
**840**	-70.04	840	-92.7	840	-46.35
**860**	-66.95	860	-90.64	860	-43.26
**880**	-72.1	880	-89.61	880	-43.26
**900**	-69.01	900	-82.4	900	-39.14
**920**	-61.8	920	-80.34	920	-36.05
**940**	-58.71	940	-77.25	940	-38.11
**960**	-55.62	960	-72.1	960	-36.05
**980**	-49.44	980	-70.04	980	-32.96
**1000**	-42.23	1000	-61.8	1000	-28.84
**1020**	-39.14	1020	-59.74	1020	-24.72
**1040**	-38.11	1040	-55.62	1040	-18.54
**1060**	-25.75	1060	-55.62	1060	-14.42
**1080**	-20.6	1080	-51.5	1080	-11.33
**1100**	-14.42	1100	-49.44	1100	0
**1120**	-9.27	1120	-38.11	1120	7.21
**1140**	0	1140	-32.96	1140	10.3
**1160**	5.15	1160	-28.84	1160	11.33
**1180**	7.21	1180	-27.81	1180	10.3
**1200**	4.12	1200	-20.4	1200	12.36
**1220**	3.09	1220	-21.63	1220	9.27
**1240**	4.12	1240	-19.57	1240	9.27
**1260**	2.06	1260	-15.45	1260	8.24
**1280**	1.03	1280	0	1280	5.15
**1300**	0	1300	7.21	1300	3.09
**1320**		1320	10.3	1320	2.06
**1340**		1340	12.36	1340	4.12
**1360**		1360	9.27	1360	1.03
**1380**		1380	8.24	1380	0
**1400**		1400	5.15	1400	
**1420**		1420	5.04	1420	
**1440**		1440	2.06	1440	
**1460**		1460	3.09	1460	

**1480**		1480	5.15	1480	
**1500**		1500	0	1500	

**Second Hydrograph, Steady flow condition; 225 min, Q_m=14 l/s**					
**0% Blockage**		**15% Blockage**		**30% Blockage**	
**Distance (mm)**	**Scour depth (mm)**	**Distance (mm)**	**Scour depth (mm)**	**Distance (mm)**	**Scour depth (mm)**
0	-54.184	0	-33.344	0	-41.68
20	-62.52	20	-50.016	20	-46.89
40	-64.604	40	-54.184	40	-50.016
60	-70.856	60	-60.436	60	-52.1
80	-62.52	80	-64.604	80	-46.89
100	-66.688	100	-67.73	100	-50.016
120	-67.73	120	-70.856	120	-52.1
140	-63.562	140	-66.688	140	-53.142
160	-67.73	160	-70.856	160	-54.184
180	-70.856	180	-71.898	180	-55.226
200	-72.94	200	-72.94	200	-56.268
220	-75.023	220	-77.108	220	-62.52
240	-76.066	240	-81.276	240	-64.604
260	-75.024	260	-83.36	260	-65.646
280	-71.898	280	-85.444	280	-64.604
300	-72.96	300	-90.654	300	-65.646
320	-75.024	320	-93.78	320	-66.688
340	-81.276	340	-92.738	340	-69.814
360	-81.276	360	-93.78	360	-72.94
380	-86.486	380	-94.822	380	-70.856
400	-88.57	400	-98.99	400	-73.982
420	-93.78	420	-102.116	420	-75.024
440	-94.822	440	-104.2	440	-75.024
460	-94.822	460	-105.242	460	-76.066
480	-95.864	480	-103.158	480	-76.066
500	-88.57	500	-102.116	500	-77.108
520	-87.528	520	-101.074	520	-75.024
540	-87.528	540	-96.906	540	-67.73

560	-86.486		560	-94.822		560	-63.562
580	-83.36		580	-89.612		580	-58.352
600	-79.192		600	-87.528		600	-56.268
620	-72.94		620	-83.36		620	-52.1
640	-70.856		640	-83.36		640	-52.1
660	-65.646		660	-81.276		660	-48.974
680	-62.52		680	-78.15		680	-43.764
700	-60.436		700	-71.898		700	-41.68
720	-59.394		720	-67.73		720	-41.68
740	-55.226		740	-62.52		740	-39.596
760	-54.184		760	-62.52		760	-42.722
780	-56.268		780	-60.436		780	-40.638
800	-50.016		800	-59.394		800	-38.554
820	-41.68		820	-56.268		820	-39.596
840	-41.68		840	-52.1		840	-36.47
860	-39.596		860	-51.058		860	-33.344
880	-38.554		880	-48.974		880	-31.26
900	-36.47		900	-44.806		900	-32.302
920	-28.134		920	-41.68		920	-29.176
940	-27.092		940	-39.596		940	-22.924
960	-20.84		960	-29.176		960	-20.84
980	-17.714		980	-20.84		980	-18.756
1000	-15.63		1000	-14.588		1000	-16.672
1020	-12.504		1020	-8.336		1020	-14.588
1040	-8.336		1040	0		1040	-12.504
1060	0		1060	2.084		1060	-10.42
1080	2.084		1080	3.126		1080	-8.336
1100	4.168		1100	5.21		1100	-5.21
1120	6.252		1120	9.378		1120	0
1140	8.336		1140	9.378		1140	2.084
1160	9.333		1160	10.42		1160	4.168
1180	9.378		1180	11.462		1180	6.252
1200	10.42		1200	13.546		1200	8.336
1220	10.57		1220	15.63		1220	8.456
1240	10.42		1240	14.588		1240	10.42
1260	9.378		1260	14.058		1260	10.42
1280	9.378		1280	12.504		1280	8.802
1300	8.054		1300	10.42		1300	8.006
1320	8.866		1320	10.42		1320	7.294
1340	7.294		1340	9.378		1340	9.378
1360	8.336		1360	8.336		1360	10.42
1380	9.378		1380	8.786		1380	10.42
1400	7.294		1400	7.294		1400	9.378

1420	4.168	1420	8.406	1420	8.336
		1440	6.252		
		1460	6.252		
		1480	5.21		
		1500	5.21		
		1520	4.928		
		1540	4.458		
		1560	4.168		
